# Dynamic transcriptome analysis and volatile profiling of *Gossypium hirsutum* in response to the cotton bollworm *Helicoverpa armigera*

**DOI:** 10.1038/srep11867

**Published:** 2015-07-07

**Authors:** Xin-Zheng Huang, Jie-Yin Chen, Hai-Jun Xiao, Yu-Tao Xiao, Juan Wu, Jun-Xiang Wu, Jing-Jiang Zhou, Yong-Jun Zhang, Yu-Yuan Guo

**Affiliations:** 1 College of Plant Protection, Northwest A & F University, Yangling, Shaanxi 712100, China; 2 State Key Laboratory for Biology of Plant Diseases and Insect Pests, Institute of Plant Protection, Chinese Academy of Agricultural Sciences, Beijing 100193, China; 3 Institute of Agro-food Science and Technology, Chinese Academy of Agriculture Sciences, Beijing 100193, China; 4 Institute of Entomology, Jiangxi Agricultural University, Nanchang 330045, China; 5 Department of Biological Chemistry, Rothamsted Research, Harpenden, AL5 2JQ, UK

## Abstract

In response to insect herbivory, plants emit elevated levels of volatile organic compounds for direct and indirect resistance. However, little is known about the molecular and genomic basis of defense response that insect herbivory trigger in cotton plants and how defense mechanisms are orchestrated in the context of other biological processes. Here we monitored the transcriptome changes and volatile characteristics of cotton plants in response to cotton bollworm (CBW; *Helicoverpa armigera*) larvae infestation. Analysis of samples revealed that 1,969 transcripts were differentially expressed (log_2_|Ratio| ≥ 2; *q* ≤ 0.05) after CBW infestation. Cluster analysis identified several distinct temporal patterns of transcriptome changes. Among CBW-induced genes, those associated with indirect defense and jasmonic acid pathway were clearly over-represented, indicating that these genes play important roles in CBW-induced defenses. The gas chromatography-mass spectrometry (GC-MS) analyses revealed that CBW infestation could induce cotton plants to release volatile compounds comprised lipoxygenase-derived green leaf volatiles and a number of terpenoid volatiles. Responding to CBW larvae infestation, cotton plants undergo drastic reprogramming of the transcriptome and the volatile profile. The present results increase our knowledge about insect herbivory-induced metabolic and biochemical processes in plants, which may help improve future studies on genes governing processes.

Plants are always exposed to attack from a large variety of herbivores. In response to insect herbivory, plants have evolved induced defense mechanisms for protection against insects. The induced defense mechanisms include direct defense such as activation of proteinase inhibitor, polyphenol oxidase, and peroxidase^1^,^2^, and indirect defense like release of a blend of terpenoids, phenylpropanoid compounds and volatile fatty acid derivatives, which is known to attract the natural enemies of the attacking insects^3^,^4^. Herbivore-induced plant defense, especially indirect defense, have large effects on the community composition at the second, third, and even higher trophic levels, resulting in a fascinating web of trophic interactions. Herbivore-induced indirect defense has been reported in an ever-increasing list of plant species, which is driving further attention to understanding of the transcriptional changes that insect herbivory trigger in plants and how such indirect defense mechanisms are orchestrated in the context of other biological processes.

The induced defense is activated only under certain conditions such as in response to herbivore damage. This relates to the recognition of attackers and the induction of signal transduction pathways, which is followed by transcriptomic changes and the induction of biosynthetic pathways^4^. Among available methods for high-throughput analysis, microarray is a powerful tool for studies of gene expression in response to herbivory, and this approach has been applied in several plant species, notably *Arabidopsis thaliana*^5^,^6^,^7^, *Nicotiana attenuata*^8^,^9^ and rice^2^,^10^. These studies demonstrate that a plant’s response to herbivore infestation is associated with large-scale changes in gene expression, and three major plant hormones, jasmonic acid (JA), salicylic acid (SA) and ethylene (ET) are essential in herbivore-induced defense responses. Other hormones, such as cytokinins, abscisic acid, gibberellins and auxin, may also play important roles in herbivore-induced defense^4^. JA is an important regulator of defense responses against insects and has been well characterized in several plants. In rice, for example, Zhou *et al.* (2011) found that genes belonging to the JA biosynthesis and signaling pathways were activated after *Chilo suppressalis* infestation, and JA levels were also increased^2^.

In cotton, infestation of chewing herbivores such as *Spodoptera exigua* or *Helicoverpa armigera* induce the release of a complex volatile blend, which might be involved in plant defense^11^,^12^. For example, *S. exigua*-damaged cotton plants specifically released several volatiles, including (*E*)-*β*-ocimene, (*E*)-*β*-farnesene, linalool, (*E*)-4,8-dimethyl-1,3,7-nonatriene (DMNT), (*E*,*E*)-4,8,12-trimethyl-1,3,7,11-tridecatetraene (TMTT) and (*Z*)-3-hexenyl acetate, which attracted parasitoid *Cotesia marginiventris*^11^. Other studies showed that with infestation of *Aphis gossypii*, cotton plants emitted a blend of defense volatiles including (*Z*)-3-hexenyl acetate, DMNT, methyl salicylate and TMTT, which can repel the cotton aphid and elicit significant antennal responses of the predatory lacewing, *Chrysoperla lucasina*^13^. In addition, application of *cis*-jasmone, a metabolite derived from the biosynthesis of JA, boosted this phenomenon^14^. The complete genome sequences of *G. raimondii*^15^,^16^ and *G. hirsutum*^17^,^18^ have been obtained recently, which will allow species of the genus *Gossypium* to be excellent model organisms for studying the molecular mechanisms of plant defense against insects. The comparative transcriptome analyses of *G. hirsutum* in response to *A. gossypii* and *Bemisia tabacci* revealed that sap-sucking insects interact with plants by suppressing the expression of phytohormonal-mediated resistance genes in order to facilitate their infestation^19^. Compared to infestation of sap-sucking insects, cotton plants responded very differently to chewing insects. In response to feeding of *Anthonomus grandis*, cotton plants activated a lot of genes involved in phytohormone signaling pathways to bolster resistance to future threats^20^.

In our previous research, we focused on tritrophic system of the chemical communication among the cotton, *H. armigera*, and the parasitoid *Microplitis mediator*. Increasing emit lots of (*E*)-*β*-ocimene was found when cotton leaves were damaged by *H. armigera* larvae, and subsequent field trials showed that parasitic wasps could perceive this terpene compound as a host location cue^12^. Recently, two terpene synthase genes of *GhTPS1* and *GhTPS2* were isolated and characterized, which were potentially involved in constitutive and herbivore-induced terpene volatiles formation in cotton^21^. In this study, we investigated the dynamic transcriptome and volatile profiling of cotton plants fed upon by larvae of a leaf-chewing herbivore cotton bollworm (CBW; *Helicoverpa armigera*). Samples from a time course of six hours to 48 hours following onset of CBW feeding were analyzed to identify target genes and key pathways involved in the activation of herbivory-induced indirect defense. In addition, the accumulation of volatile organic compounds (VOCs), which represent changes in cotton plant phenotype, was also monitored following CBW infestation.

## Results

### Global transcriptome changes of cotton leaves in response to CBW infestation

**Figure 1:**
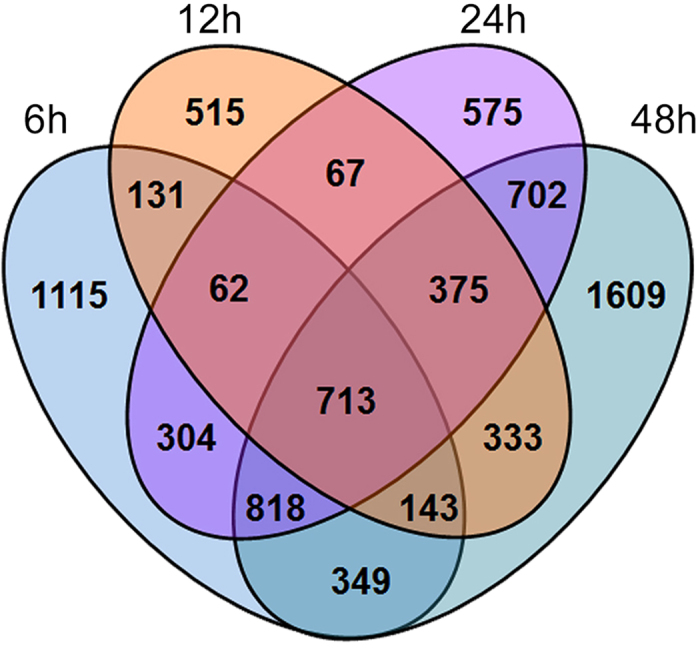
Comparative analysis of changes in the cotton leaf transcriptome in response to cotton bollworm at different time-points. Genes associated with a *q*-value of less than or equal to 0.05 for at least one time point were used to construct the Venn diagram.

**Figure 2:**
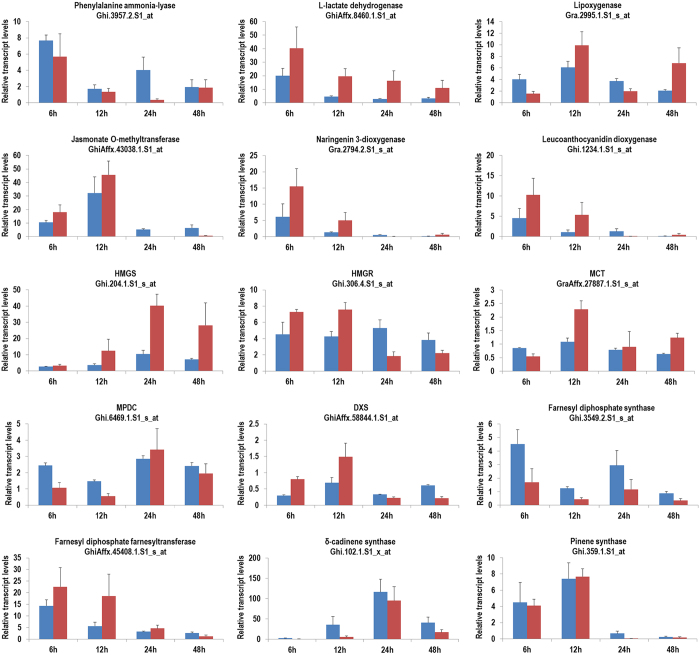
Comparison of qRT-PCR (red bar) and microarray (blue bar) expression data for selected gene. HMGS, hydroxymethylglutaryl-CoA synthase; HMGR, hydroxymethylglutaryl-CoA reductase (NADPH); MCT, 2-C-methyl-D-erythritol 4-phosphate cytidylyltransferase; MPDC, mevalonate diphosphate decarboxylase; DXS, 1-deoxy-D-xylulose-5-phosphate synthase.

The number of genes associated with a *q*-value of less than or equal to 0.05 at each time point was found to range from 2,339 (1184 up- and 1155 down-regulated) after 12 h of CBW feeding to 5,042 (2,421 up- and 2,621 down-regulated array elements) after 48 h of herbivory (Supplementary Table S1 and S2). A Venn diagram was constructed to identify commonly and exclusively regulated genes in response to CBW infestation over the 48 h time course (Fig. 1). We observed that 9% genes were commonly expressed in response to CBW feeding over the 48 h time course. In addition, many genes were exclusively expressed at 6 h after the onset of CBW infestation, which indicates that these genes are likely to play roles in the herbivory-induced early signaling events. To evaluate the reproducibility of the microarray data, 15 genes were selected for qPCR analysis (Fig. 2). These included genes related to phytohormone biosynthesis and secondary metabolism, including one phenylpropanoid-biosynthesis gene, one ET-biosynthesis gene, two JA-biosynthesis genes, two flavonoid-biosynthesis genes and nine terpenoid-biosynthesis genes. For most of these genes, expression patterns tested by qPCR were highly consistent with the results of microarray analysis, indicating that the microarray assay can meet the further research requirements to identify target genes and key pathways involved in the activation of herbivory-induced indirect defense.

**Figure 3:**
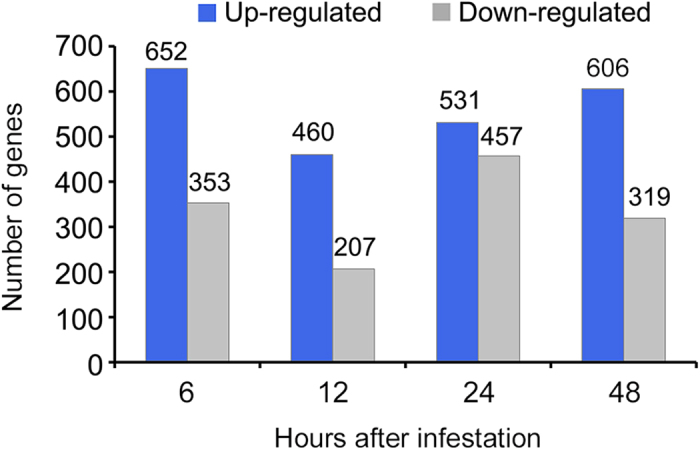
Number of the up-regulated and down-regulated DEGs for the time series of 6, 12, 24 and 48 h of *Helicoverpa armigera* feeding. The DEGs are those displaying a change of more than four-fold with a *q*-value of less than or equal to 0.05 for at least one time point.

**Figure 4:**
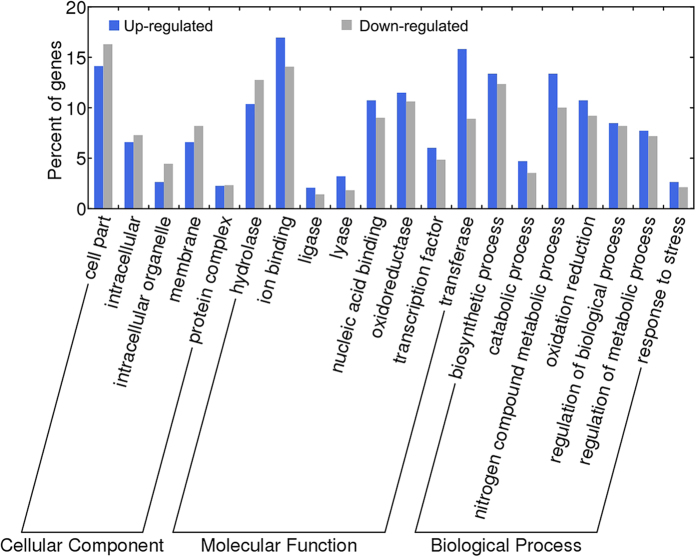
Distribution of DEGs in the cotton leaf in response to CBW based on GO functional categories. The DEGs are those displaying a change of more than four-fold with a *q*-value of less than or equal to 0.05 for at least one time point.

Genes associated with a cut-off of *q* ≤ 0.05 and the value of log_2_|Ratio| ≥2 considered to show significantly different expression. A total of 1,969 transcripts were differentially expressed at one or more time points (Supplementary Table S1). Among these 1,969 transcripts, 1,120 were significantly up-regulated, 846 were significantly down-regulated and only three were mixed (Supplementary Table S1). Many of these genes were differentially expressed at 6 h after the onset of CBW infestation, and the numbers of differentially expressed genes (DEGs) decreased thereafter. At all time points, more genes were up-regulated than down-regulated (Fig. 3). Gene functions were classified using Gene Ontology (GO) analysis. In total, twenty GO terms were attributed to one of the three GO ontologies (cellular component, molecular function and biological process) (Fig. 4). In the cellular components GO ontology, the top term was cell part. Meanwhile, most of the genes were classified into the function ontology of ion binding and transferase activity. The GO analysis also showed that both the biosynthetic process and nitrogen compound metabolic process GO categories were most abundant in the biological process ontology. Additionally, further analysis of GO clustering of commonly and exclusively expressed genes at four time points was carried out (Supplementary Figure S1). For GO clustering of catalytic activity, exclusively expressed genes at 48 h after CBW infestation accounted for the largest proportion followed by exclusively expressed genes at 6 h. Their corresponding percentages were higher in comparison to the percentage of commonly expressed genes at four time points, suggesting that most of plant responses to CBW were in common at 6 h and 48 h after infestation, although with an enhanced reaction at 48 h (Supplementary Figure S1A). Also, exclusively expressed genes at 48 h after CBW infestation have the largest proportion of DEGs involved in transcription factor activity, biological process, metabolic process and biosynthetic process (Supplementary Figure S1B and S1C). These GO analysis provided valuable clues to investigate the specific processes and molecular functions of transcriptome changes of cotton leaves in response to CBW infestation.

### Temporal patterns of the cotton transcriptome

**Figure 5:**
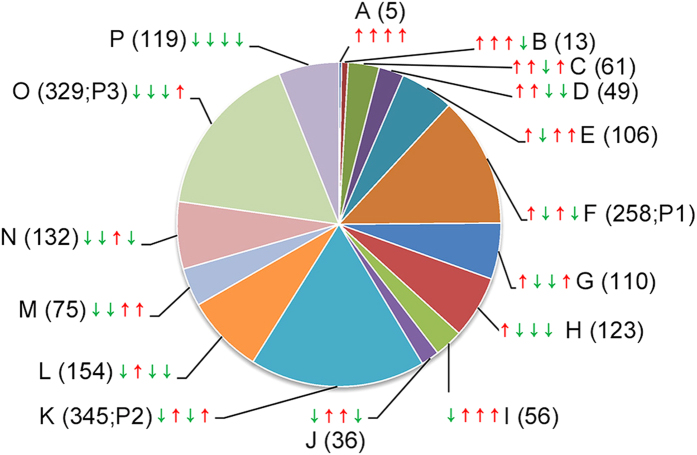
Clusters of DEGs defined by the K-means algorithm and grouped based on the dynamics of expression changes during the time course. Red arrows: up-regulated expression at one time point relative to the control or the previous time point; green arrows: down-regulated expression at one time point. Arrows are followed by cluster designation and the number of genes in each cluster. P1, P2 and P3 are the top three clusters.

Among 7,811 transcripts associated with a *q*-value of less than or equal to 0.05 for at least one time point, 1,971 were placed into 16 expression clusters based on the temporal patterns in their expression profiles as identified by K-means clustering (Fig. 5). Although 597 genes displayed a rapid transient up-regulation within 6 h upon onset of herbivory (Clusters E - H), a majority of 110 genes peaked at 12 h (clusters C and cluster D). Another group of DEGs peaked at 24 h (cluster B), whereas five genes were up-regulated early during the treatment and maintained high expression levels relative to control plants (cluster A). Similarly, many down-regulated genes displayed transient expression profiles (Clusters I - O), although 119 down-regulated genes maintained lower expression levels over the entire time course analyzed (cluster P). In order to produce a global description of gene functions enriched in the top three clusters of similarly regulated transcripts, we generated an overview of GO annotation (Supplementary Table S3).

### Expression profiles of genes associated with phytohormone and transcription factors

**Figure 6:**
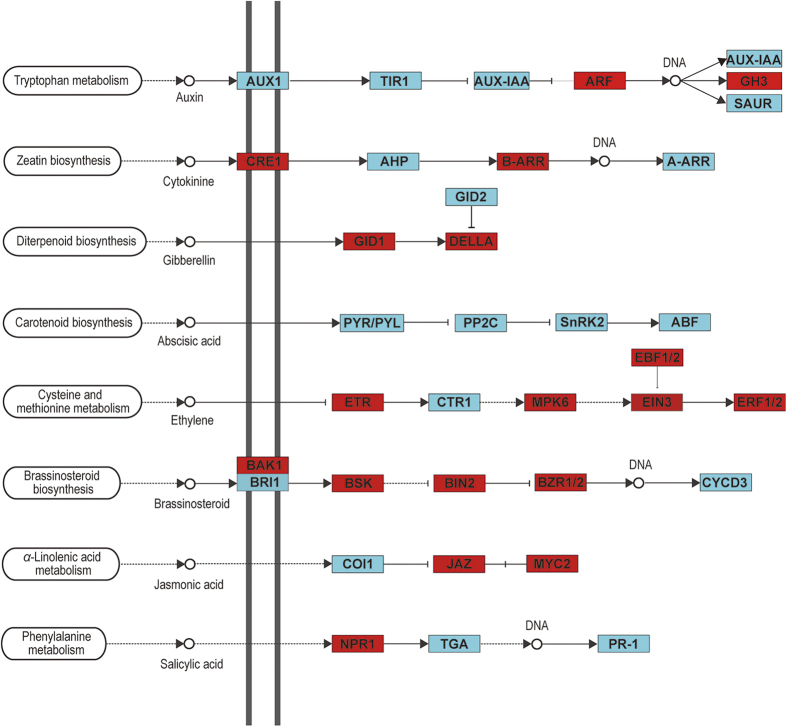
Examples of KEGG pathways found for transcripts associated with phytohormone signaling. Each box shows enzymes involved in each section of the pathway. Genes highlighted in red were up-regulated, and those in green were down-regulated.

Based on the central role of phytohormones in plant defense responses to insect herbivore attack, we investigated the profiles of transcripts associated with signaling molecules and phytohormones: auxin, cytokinin, abscisic acid (ABA), brassinosteroids (BR), gibberellic acid (GA), ET, JA, and SA (Fig. 6, Supplementary Figure S2 and Supplementary Table S4 and S5).

Twenty six JA biosynthesis genes and 12 JA signaling genes responded to CBW feeding, and all the genes were up-regulated. These genes included lipoxygenase (LOX), allene oxide synthase (AOS), allene oxide cyclase (AOC), 12-oxophytodienoate reductase (12-OPR), jasmonate O-methyltransferase (JMT), jasmonate ZIM domain-containing protein (JAZ) and transcription factor MYC2 (MYC2). Almost all of these genes were up-regulated at early course during the treatment, maintained high expression levels than control plants. In addition, two coronatine-insensitive protein 1 (COI1) genes were modestly down-regulated by CBW feeding as shown in Fig. 6.

Similarly, CBW feeding enhanced the expression of genes associated with ET and abscisic acid. Eight ET biosynthesis genes and six ET signaling genes, consisting of two S-adenosylmethionine synthetases, two 1-aminocyclopropane-1-carboxylate synthases and four aminocyclopropanecarboxylate oxidases, were up-regulated rapidly within 6 h after the onset of CBW feeding and stayed up-regulated. In contrast, only one S-adenosylmethionine synthetase gene was down-regulated at the 24 h time point and two serine/threonine-protein kinase CTR1 genes were modestly down-regulated over the time course. Six ABA biosynthesis genes were up-regulated, and an ABA-signaling gene was down-regulated.

Seven genes encoding enzymes involved in GA biosynthesis were responsive to CBW feeding. Among these genes, three were up-regulated and one was down-regulated. Fourteen DEGs associated with GA signaling were also over-represented in the CBW affected transcriptome. Among these 14 genes, ten genes, including four gibberellin receptor GID1 (GID1) genes and six DELLA proteins (DELLA), were up-regulated, and four genes were down-regulated at one or more time points. In addition, two F-box protein GID2 (GID2) genes were modestly down-regulated by CBW feeding.

Genes associated with auxin, BR, and cytokinin exhibited complex regulatory patterns: eleven auxin biosynthesis and signaling genes, seven BR genes and eight cytokinin genes were up-regulated, and ten auxin genes, four BR genes and five cytokinin genes were down-regulated.

In contrast to the JA- and ET-related transcriptome signatures, almost all of the genes related to SA biosynthesis and signaling were down-regulated late in the feeding experiment, except for one phenylalanine ammonia-lyase (PAL) that was up-regulated within 6 h after onset of insect feeding. Overall, our results highlight the importance of jasmonate in herbivore induced signaling, and may suggest additional roles for ET and gibberellin.

Furthermore, we analyzed the expression profiles of genes known or predicted to be involved with transcription factors (TFs), in order to gain insights into possible signaling processes elicited by CBW feeding (Supplementary Table S6). Among the 545 TFs represented on the array, 212 were differentially expressed at one or more time points, with 144 being up-regulated and 68 down-regulated. Among these, 102 TFs were differentially expressed at two or more time points, 20 of which were down-regulated and 82 were up-regulated. Among the 20 down-regulated TFs, bZIP, CO-like and HD-ZIP type factors form the dominant group. TFs that were up-regulated by CBW feeding predominantly belong to the WRKY, ERF, MYB, NAC and C2H2 families. Among the 82 up-regulated genes, the largest family was WRKY (25 genes), followed by ERF (13 genes).

### Expression profiles of defense-related genes induced by CBW larvae

Next, the expression of defense-related gene annotated within the categories ‘defense response/response to biotic stimulus’, ‘response to stress’, ‘defense mechanisms’ and ‘disease resistance protein’, which are reported to be involved in plant defense in response to various pathogens and insects were systematically examined. The cluster of stress-related genes included 163 genes, of which 66 (40%) were differentially expressed in response to CBW feeding (Supplementary Table S7). Most of these CBW-affected stress-related genes were strongly up-regulated within 6 h after the onset of CBW feeding, including putative disease resistance response protein, class 10 PR protein, *δ*-cadinene synthase and salicylate O-methyltransferase. Ten genes showed up-regulation of transcript abundance mainly at late time-points, including dirigent-like proteins that have been proposed to play a role in lignin synthesis^22^. Meanwhile, CBW feeding suppressed some of the certain genes that responded to biotic stimuli, including GLP3, putative major latex protein and pathogenesis-related protein 1 (PR1).

**Figure 7:**
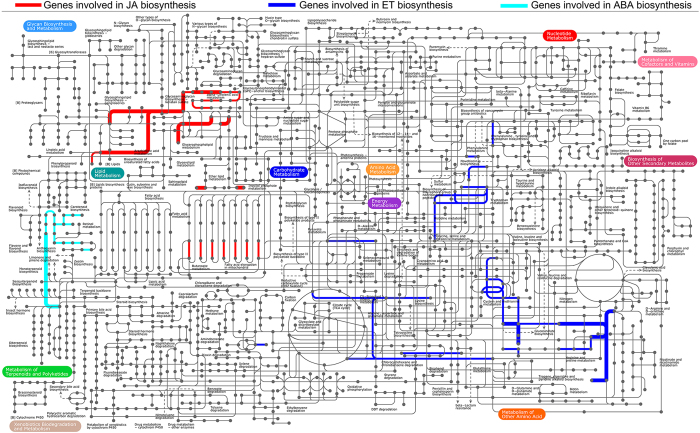
Secondary metabolite pathways expressed in *Gossypium hirsutum* after feeding by the cotton bollworm *Helicoverpa armigera*. The map was generated with iPath ( http://pathways.embl.de), a web-based tool for the visualization of metabolic pathways.

**Figure 8:**
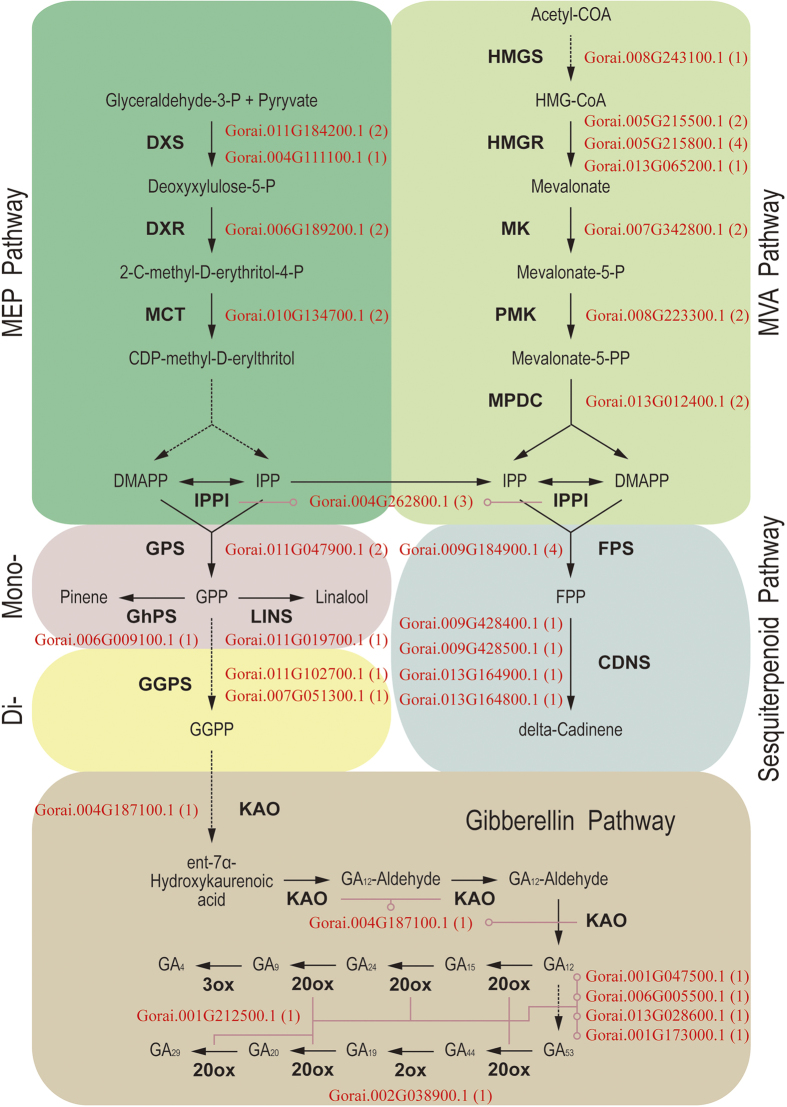
Expression patterns of cotton bollworm-induced genes involved in the terpenoid biosynthetic pathways. Gene ID is followed in parentheses by the number of EST. Solid arrows represent established biosynthetic steps, whereas broken arrows illustrate the involvement of multiple enzymatic reactions. 2ox, GA 2-oxidase; 3ox, GA 3-oxidase; 20ox, GA 20-oxidase; CDNS, cadinene synthase; DMAPP, dimethylallyl pyrophosphate;DXS, 1-deoxy-D-xylulose 5-phosphate synthase; DXR, 1-deoxy-D-xylulose 5-phosphate reductoisomerase; FPP, farnesyl pyrophosphate; FPS, FPP synthase; GA, gibberellin; GGPP, geranylgeranyl pyrophosphate; GGPS, GGPP synthase; GPP, geranyl pyrophosphate; GPS, GPP synthase; HMG-CoA, hydroxymethylglutaryl-CoA; HMGR, HMG-CoA reductase; HMGS, HMG-CoA synthase; IPI, isopentenyl pyrophosphate isomerase; IPP, isopentenyl pyrophosphate; KAO, ent-kaurenoic acid oxidase; LINS, linalool synthase; MCT, 2-C-methyl-D-erythritol 4-phosphate cytidylyltransferase; MK, mevalonate kinase; MPDC, mevalonate diphosphate decarboxylase; PMK, phosphomevalonate kinase.

We further analyzed the expression patterns of genes of secondary metabolite pathways that are known to be affected by herbivory, specifically phenylpropanoid, flavonoid and terpenoid metabolism (Fig. 7 and Supplementary Table S8 and S9). Many transcripts from these pathways were differentially expressed upon CBW feeding. The largest subgroup was phenylpropanoid biosynthesis (16 up- and 12 down-regulated genes), followed by flavonoid biosynthesis genes, most of which (21 of 23) were down-regulated. For the phenylpropanoid pathway, the first committed step is catalyzed by L-phenylalanine ammonia lyase (PAL), a well-known and widely distributed enzyme. Microarray analysis showed that one PAL was up-regulated within 6 h after the onset of CBW feeding, and three other PALs displayed repressed expression at the 48 h time point. CBW feeding induced two genes that have been functionally characterized as encoding enzymes of the flavonoid pathway, but many of the genes in this pathway were represented. To elucidate the molecular basis for the biosynthesis of volatiles involved in indirect defenses of cotton to CBW, we mainly addressed terpenoid metabolism (Fig. 8). Three genes encoding the crucial enzyme of the non-mevalonate pathway, 1-deoxy-D-xylulose 5-phosphate synthase were modestly down-regulated by CBW feeding. In contrast, two hydroxymethylglutaryl-CoA reductases of the mevalonate pathway were up-regulated. Consistently, a geranyl diphosphate synthase was down-regulated, and a farnesyl diphosphate synthase was up-regulated. In addition, two monoterpene synthases, including a previously characterized pinene synthase, and four cadinene synthases, a sesquiterpene synthases and the catalyst for cotton phytoalexin biosynthesis, were up-regulated.

### Cotton VOCs changes in CBW-infested plants

**Figure 9:**
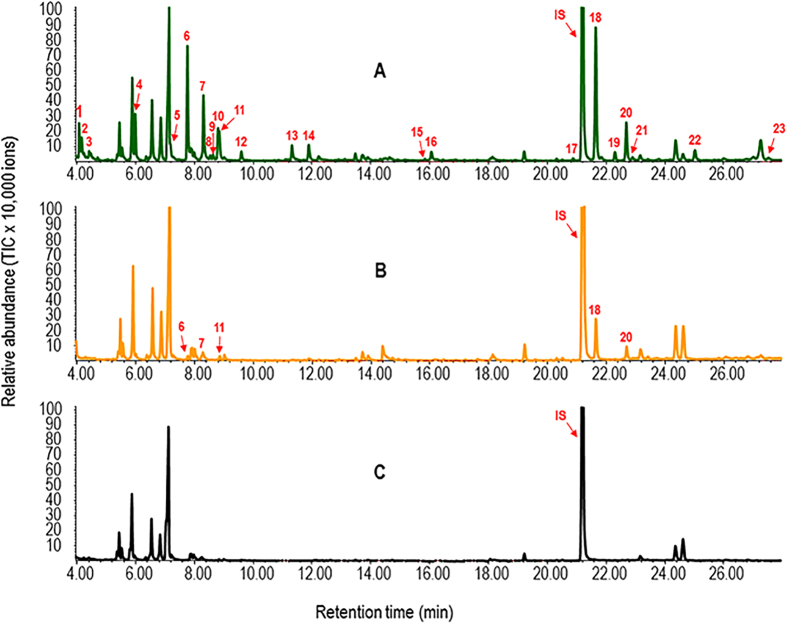
Representative chromatograms of head-space volatile compounds from cotton plants that were infested by cotton bollworm for 48 h (**A**), untreated cotton plants (**B**), or an empty container (a pot of soil without plants) (**C**). Detail information of volatile compounds is shown in Table 1. IS, internal standard (ethyl caprate).

Based on analysis of the expression patterns of genes encoding enzymes responsible for the biosynthesis of volatiles, we further compared volatile profiles between treatment and control. Representative chromatograms of head-space volatile compounds from cotton bollworm-damaged cotton plants are shown in Fig. 9. Lipoxygenase-derived green leaf volatiles, including (*Z*)-3-hexenol, (*Z*)-3-hexenyl acetate and (*E*)-2-hexenyl acetate, and a number of terpenoids such as limonene, caryophyllene, and DMNT were emitted transiently in relatively large amounts during early stages of damage. As damage progressed, however, there was increased production of several other terpenes including (*E*)-*β*-ocimene, linalool, *δ*-cadinene and TMTT, which had been released only in relatively small amounts during early stages of damage (Fig. 9; Table 1). Cotton VOCs changes in CBW-infested plants were basically consistent with the results of transcriptome analysis, indicating cotton plants undergo drastic reprogramming of the transcriptome and the volatile profile in response to CBW larvae feeding.

## Discussion

### CBW infestation induces temporal transcriptomic changes in cotton plants

CBW larvae infestation induces substantial overall changes in the cotton leaf transcriptome, with 1,120 genes that were significantly induced and 846 genes that were significantly repressed. Despite continuous feeding for 48 h, the majority of DEGs displayed a rapid transient up-regulation within 6 h upon onset of herbivory and transcript levels were no longer increased at late time points (Clusters E - H, Fig. 5). The impact of insect attack on large-scale transcriptome changes has been studied in a few plant species. Existing reports support the general notion that insect feeding induces massive changes in the host plant transcriptome^2^,^5^,^6^, including the massive reprogramming of primary and secondary metabolic processes and rapid changes in signaling and other regulatory processes. These findings are well supported by our analysis of the effect of CBW feeding on the cotton transcriptome. The present study establishes a signature of CBW-induced changes to the signaling transcriptome of cotton leaves.

### Cotton plants reprogramme primary and secondary metabolism in response to CBW infestation

Many secondary metabolites, such as phenylpropanoids, flavonoid and terpenoids confer resistance to herbivory or serve as communication signals between plants and insects. Most genes involved in phenylpropanoid and terpenoid metabolism were up-regulated, whereas almost all genes related to flavonoid metabolism were down-regulated. These results suggest that CBW infestation mainly activated the phenylpropanoid and terpenoid pathways.

Terpenoids are the most common group of secondary metabolites, which can directly repel herbivores and also attract natural enemies of the attacking insects. In cotton, feeding of chewing herbivores such as *S. exigua* or *Helicoverpa zea* and piercing-sucking herbivores such *Lygus hesperus* induces the release of a complex volatile blend including *β*-myrcene, (*E*)-*β*-ocimene, DMNT and (*E*)-*β*-caryophyllene, which increase the foraging efficiency of predators and parasitoids^23^,^24^,^25^. Here we found that CBW infestation increased both the mRNA levels of many genes involved in terpenoid metabolism and the levels of the blend of cotton terpenoid volatiles. While, key genes of non-mevalonate pathway were modestly down-regulated in cotton plants damaged by CBW, key genes in the mevalonate pathway were found to be up-regulated. The mevalonate pathway is the biosynthetic pathway of gossypol and its derivates, which acts as an important phytoalexin and provides constitutive and inducible resistance against a variety of pests and diseases^26^,^27^. Moreover, both the mRNA levels of four cadinene synthases and the levels of the corresponding volatile, a precursor of hemigossypol, were increased. Therefore, the mevalonate pathway is likely to be the main terpenoid pathway response to CBW-infestation in cotton.

In addition, during CBW feeding, 28 genes involved in photosynthesis were differentially expressed, of which almost all (27 genes) were down-regulated, indicating that cotton plants reprogram both primary and secondary metabolism in response to CBW infestation, probably as a strategy to make more energy available for the synthesis of defense materials and the elicitation of the defense response^28^. Changes in metabolism may be a general phenomenon in plant responses to herbivores. Numerous studies have demonstrated that herbivory can results in down-regulation of primary metabolic processes while simultaneously activating defense-related processes including secondary defense metabolism.

### JA, ET and GA function in CBW-induced defense signaling

Phytohormones act as central players in plant defenses in the context of growth and development, as demonstrated by the fact that genes involved in phytohormone biosynthesis and signaling pathways were differentially expressed in response to CBW infestation (Fig. 6 and Supplementary Table S4 and S5). The phytohormones auxin, BR and cytokinin all exhibited a complex regulatory pattern following CBW feeding. Our results indicated that following herbivore infestation, these hormones tend to maintain their homeostasis to ensure they meet the basic needs for plant growth and survival. In contrast, most genes involved in the JA, ET and GA biosynthesis and signaling pathways were induced, suggesting that JA, ET and GA may play major roles in the regulation of signaling networks involved in CBW-induced defense responses.

JA, derived from *α*-linolenic acid via one branch of the octadecanoid pathway, is an important regulator of defense responses against pathogens and chewing insects. We observed the activation of many genes involved in the JA biosynthesis and signaling pathways, such as LOXs, AOS, AOC, JMT, JAZ and MYC2 TF (Supplementary Table S5). These findings are similar to those reported for other dicotyledonous and monocotyledonous plants^2^,^6^,^29^. Recent discoveries have shown that JAZ proteins are crucial regulators of the jasmonate hormonal response^30^. We found that all eight cotton JAZ genes were up-regulated. This indicates that in cotton, JA signal transduction may act through a mechanism similar to that in other plants.

Moreover, chemical analysis of plant volatiles showed that emissions of green leaf volatiles, which are synthesized via the other branch of octadecanoid pathway, displayed a rapid transient within 12 h upon onset of herbivory. Green leaf volatiles have far-reaching effects on a plant’s interactions with other plants. For example, *Manduca sexta* infestation elicited a rapid isomeric change in the green leaf volatiles release of *N. attenuata* plants. This change increased the predation rate of the generalist predator^31^.

ET is a major constituent of the blend of defense signals and functions as an important modulator in plant responses to biotic and abiotic stress. Here we found that CBW infestation enhanced the expression of many genes involved in ET biosynthesis and signaling. These results suggest that the ET-mediated signaling pathway was also activated and exerts an active role in CBW-induced defense responses. The expression levels of ET-related genes were also changed in cotton infested by the sap-sucking insects such as aphid and whitefly^19^. Thus, ET is a general signal that modulates the cotton plant’s defense against both chewing and sap-sucking herbivores.

Moreover, most ABA- and GA-related genes were also activated during the infestation by CBW. Although the roles of ABA and GA in plant-insect interactions remain unclear, their importance in this area has recently been demonstrated^32^. Furthermore, recent reports have shown that genes involved in ABA and GA biosynthesis and signaling pathways were induced in sorghum infested with greenbug *Schizaphis graminum*^33^ and in cotton infested with aphid and whitefly^19^.

Numerous studies have demonstrated that crosstalk between JA and SA is mutually antagonistic, and these findings support the general notion that chewing caterpillars may induce JA responsive genes that influences the activity of primary SA responsive genes activated by pathogens and sap-sucking whiteflies or aphids^34^. This theory is well supported by our analysis of the cotton transcriptome affected by CBW infestation. Here we found that in contrast to the high induction of jasmonate-related genes, some genes related to SA biosynthesis were down-regulated, including three genes for PAL, the crucial enzyme that contribute to SA biosynthesis, and two genes for the SA signaling protein PR1. It is possible that the JA signaling pathway elicited by CBW may suppress SA-related genes.

### CBW feeding regulates transcription factors

Recent studies have implicated TF genes in insect-induced resistance^35^. Here we found that 102 TF genes belonging to 23 TF families were responsive to CBW infestation at two or more time points. This large number of TF genes may reflect the complexity of defense regulation and a drastic transcriptional reprogramming in the cotton plants in response to CBW infestation. Among the 82 up-regulated TF genes, four gene families, WRKY, ERF, MYB, and NAC, showed relatively high representation (58 genes). All of these families play important roles in plant responses to abiotic and biotic stress, as well as in plant growth and development. For example, the role of certain WRKY genes in the regulation of plant response to herbivory has been demonstrated through genetic modification^36^,^37^. Moreover, much evidence has shown that NAC genes play important roles in plant responses to fungal infection and ABA and JA treatment^38^.

## Methods

### Plants and insects

Cotton seeds (*Gossypium hirsutum* cv. CCRI12) were sown in plastic pots (height, 14 cm; diameter, 16 cm). Seedlings were grown in a growth chamber under 29/25 °C temperature and a 16:8 h light:dark cycle, and water was added every two days. All plants were used in experiments at the 6–7 fully expanded true leaf stage, which occurred 5–6 weeks after sowing. To obtain enough plant materials for RNA isolation, each treatment consisted of three plants grown together in one pot. A field population of *H. armigera* was originally collected from Xinxiang County, Henan Province of China in 1996^39^. Insects were reared on an artificial diet and maintained at 27 ± 2 °C, 75 ± 10% relative humidity, and 14:10 h light:dark in the laboratory.

### Plant treatments

Thirty-six *H. armigera* larvae (third instars) were placed on a group of three cotton plants. In order to prevent the escape of larvae, we used a nylon mesh bag (30 × 40 cm, 30 mesh) to cover each treatment. Samples for each time point maintained separately till to be harvested. Undamaged plants maintained under the same conditions were used as controls. Cotton leaves from control plants and plants exposed to *H. armigera* were harvested at 6 h, 12 h, 24 h, and 48 h after onset of herbivory. For each treatment group and time point, cotton leaves were harvested from the three plants per treatment group and flash frozen in liquid nitrogen. For each time point, three replicate treatments and controls were performed.

### RNA isolation, cDNA labeling and microarray hybridization

Total RNA extractions were performed using a modified hot borate method^40^. The purity and quantity of the obtained RNA was determined using a Nanodrop ND 1000 instrument (Nanodrop Technologies, Wilmington, DE, USA). RNA integrity was analyzed via formaldehyde agarose gel electrophoresis. All procedures for RNA labeling and microarray hybridization were performed as described previously^41^. The microarray data were deposited at GEO (Gene Expression Omnibus) at the National Center for Biotechnology Information (NCBI) http://www.ncbi.nlm.nih.gov/geo/ with the accession number GSE62158.

### Quantitative PCR analysis

RNA extracted as described above was converted to cDNA using the FastQuant RT Kit (Tiangen, Beijing, China) according to the manufacturer’s instructions. Real time quantitative PCR (qPCR) analyses were carried out following the procedures described by Gu *et al.* (2013; cited in Ref. 42). GhACT4 and GhPP2A1 were used as reference genes as the expression levels were most stable in cotton leaves^43^. Specific primer pairs were designed with Primer 3.0 ( http://frodo.wi.mit.edu/) (Supplementary Table S10). For each time point, three biological replicates, as described above, were analyzed using qPCR.

### Bioinformatics analysis

The expression patterns were clustered using Cluster software^44^. The pathways were annotated based on the KEGG database^45^ using BLASTX (e ≤ 1e^−5^). KEGG mapper and iPath tools were used for the plant-insect interaction pathway and the phytoalexin biosynthesis pathway analyses, respectively^45^,^46^. Gene Ontology was identified in the GO database through Blast2GO software^47^ using the default parameters.

### Collection and identification of VOCs

Twelve *H. armigera* larvae were placed on one cotton plant following the method mentioned above. Pots containing one *H. armigera*-exposed or control plant were randomly placed within a glass jar (25 cm in diameter × 60 cm high) at 6 h, 12 h, 24 h, or 48 h after onset of herbivory. The collection system similar to that described by Yu *et al.* (2010; cited in Ref. 12) was used to collect cotton plant volatiles. The container was sealed with a glass lid that had an air inlet and an air outlet. The container then was tightly sealed with metal clamps on the lid. Air, purified by passage through an activated charcoal filter, was actively pumped through the container at a flow rate of 1.5 ml min^−1^ with a vacuum pump (Beijing Institute of Labor Instrument, Beijing, China). Volatiles were collected for 12 h on 50 mg of 60/80 mesh Tenax-TA (Shanghai ANPEL Scientific Instrument Company, Shanghai, China) in a 8 mm diameter glass tube, which was directly connected to the outlet. All connections were made with Teflon tape. The collection of volatiles for each treatment was repeated 3–5 times.

After collection, volatiles were extracted with 300 μl of hexane (Fisher, Fairlawn, NJ), in which 7.451 μg of ethyl caprate (Sigma-Aldrich, Oakville, Canada) was individually added as an internal standard. A sample volume of 1 μl was taken for gas chromatography-mass spectrometry (GC-MS) analysis as described previously^21^. Briefly, the instrument was equipped with a HP-5 capillary column (30 m × 0.25 mm i.d. × 0.25 μm film thickness; Agilent, Santa Clara, CA). Samples were injected without split at an initial oven temperature of 40 °C (1 min hold), followed by a two-step temperature increase, first to 130 °C (at a rate of 4 °C min^−1^, 5 min hold) and then to 250 °C (at a rate of 10 °C min^−1^, 5 min hold). Products were identified by comparison of their retention times and mass spectra with those of authentic standards (Sigma-Aldrich, Oakville, Canada) analyzed under the same conditions.

## Additional Information

**How to cite this article**: Huang, X.-Z. *et al.* Dynamic transcriptome analysis and volatile profiling of *Gossypium hirsutum* in response to the cotton bollworm Helicoverpa armigera. *Sci. Rep.* **5**, 11867; doi: 10.1038/srep11867 (2015).

## Supplementary Material

Supplementary Information

Supplementary Information

## Figures and Tables

**Table 1:** Proportions (% of internal standard compound) of volatile compounds emitted from control (C) or CBW-damaged plants (CBW) over the full time course.

**Compound**	**RT**	**6h-C**	**6h-CBW**	**12h-C**	**12h-CBW**	**24h-C**	**24h-CBW**	**48h-C**	**48h-CBW**
1. (*E*)-2-Hexenal	4.078	**nd**	**nd**	**nd**	**4.39 ± **2.25	**nd**	**1.27 ± **0.65	**nd**	**3.04 ± **2.08
2. (*Z*)-3-Hexenol	4.164	**nd**	**nd**	**4.11 ± **3.60	**32.28±** 27.71	**nd**	**1.03 ± **0.17^*^	**nd**	**1.86 ± **0.80
3. (*E*)-2-Hexenol	4.418	**nd**	**1.61 ± **0.86	**nd**	**1.97 ± **0.54^*^	**nd**	**0.77 ± **0.27^*^	**nd**	**1.10 ± **0.21^**^
4. *α*-Pinene	5.976	**nd**	**nd**	**1.80 ± **0.72	**6.09 ± **1.38	**nd**	**10.39 ± **8.79	**nd**	**6.36 ± **2.47^*^
5. *β*-Pinene	7.171	**nd**	**nd**	**nd**	**nd**	**nd**	**0.88 ± **0.69	**nd**	**1.24 ± **0.09^**^
6. *β*-Myrcene	7.751	**nd**	**nd**	**nd**	**17.79 ± **6.49^*^	**nd**	**29.96 ± **27.35	**0.39 ± **0.05	**19.48 ± **4.27^**^
7. (*Z*)-3-Hexenyl acetate	8.296	**nd**	**40.59 ± **12.89^*^	**44.69 ± **28.04	**50.22 ± **23.92	**nd**	**26.89 ± **17.30	**0.66 ± **0.32	**19.10 ± **4.77^*^
8. Hexyl acetate	8.519	**nd**	**nd**	**nd**	**0.74 ± **0.24^*^	**nd**	**2.58 ± **0.84^*^	**nd**	**1.35 ± **0.70
9. 2-Hexenyl acetate	8.625	**nd**	**3.57 ± **0.47^**^	**nd**	**nd**	**nd**	**2.54 ± **0.78^*^	**nd**	**1.01 ± **0.24^*^
10. 1-Decyne	8.795	**nd**	**nd**	**nd**	**nd**	**nd**	**4.20 ± **2.44	**nd**	**12.55 ± **6.48
11. Limonene	8.835	**nd**	**4.64 ± **2.76	**4.52 ± **1.70	**4.74 ± **0.79	**1.19 ± **0.02	**7.71 ± **6.11	**0.57 ± **0.08	**4.06 ± **0.73^**^
12. *β*-Ocimene	9.590	**nd**	**nd**	**nd**	**4.68 ± **2.56	**nd**	**9.44 ± **7.42	**nd**	**4.82 ± **2.58
13. Linalool	11.316	**nd**	**nd**	**nd**	**3.14 ± **0.98^*^	**nd**	**7.30 ± **4.15	**nd**	**10.40 ± **6.48
14. DMNT	11.888	**nd**	**7.92 ± **0.77^**^	**nd**	**11.25 ± **5.58	**nd**	**22.35 ± **13.91	**nd**	**12.70 ± **8.31
15. Hexenyl valerate	15.844	**nd**	**nd**	**nd**	**nd**	**nd**	**0.58 ± **0.21^*^	**nd**	**1.02 ± **0.66
16. 3-Hexenyl isovalerate	16.065	**nd**	**nd**	**nd**	**nd**	**nd**	**3.83 ± **0.62^**^	**nd**	**1.93 ± **0.13^**^
17. *β*-Elemene	20.876	**nd**	**nd**	**nd**	**0.47 ± **0.02^**^	**nd**	**0.45 ± **0.05^**^	**nd**	**1.05 ± **0.56^*^
18. *β*-Caryophyllene	21.658	**nd**	**5.40 ± **2.26^*^	**11.35 ± **3.07	**21.11 ± **14.80	**3.60 ± **.83	**24.62 ± **14.42	**9.30 ± **1.17	**30.32 ± **6.20^*^
19. *α*-Guaiene	22.308	**nd**	**nd**	**nd**	**1.14 ± **0.41^*^	**nd**	**1.70 ± **0.89	**nd**	**1.60 ± **0.54^*^
20. *α*-Humulene	22.694	**nd**	**nd**	**3.47 ± **0.98	**7.08 ± **4.50	**1.27 ± **0.17	**7.81 ± **4.61	**2.72 ± **0.31	**13.57 ± **3.25^*^
21. Unk sesquiterpene	22.902	**nd**	**nd**	**nd**	**0.83 ± **0.08^**^	**nd**	**nd**	**nd**	**2.29 ± **1.63
22. *δ*-Cadinene	25.021	**nd**	**nd**	**nd**	**nd**	**nd**	**1.10 ± **0.27^*^	**nd**	**4.13 ± **0.73^**^
23. TMTT	27.510	**nd**	**nd**	**nd**	**0.99 ± **0.42^*^	**nd**	**1.81 ± **0.40^**^	**nd**	**2.29 ± **1.50

nd, not detected. Asterisks indicate significant differences in CBW-treated cotton compared to the corresponding control by Student’s t-test at each time point (*P < 0.05, **P < 0.01).
