# Supplementary material for: Dynamic transcriptome analysis and volatile profiling of Gossypium hirsutum in response to the cotton bollworm Helicoverpa armigera
Source: Sci Rep. 2015 Jul 7;5:11867. doi: 10.1038/srep11867 (PMC4493570; doi:10.1038/srep11867)
Supplement: Supplementary Information [file srep11867-s1.pdf]

SUPPLEMENTARY INFORMATION

Dynamic transcriptome analysis and volatile profiling of *Gossypium hirsutum* in response to the cotton bollworm *Helicoverpa armigera*

Xin-Zheng Huang[1,2]*, Jie-Yin Chen[3]*, Hai-Jun Xiao[4], Yu-Tao Xiao[2], Juan Wu[2], Jun-Xiang Wu[1]†, Jing-Jiang Zhou[5] ,Yong-Jun Zhang[2]† & Yu-Yuan Guo[2]

[1]College of Plant Protection, Northwest A & F University, Yangling, Shaanxi 712100, China, [2]State Key Laboratory for Biology of Plant Diseases and Insect Pests, Institute of Plant Protection, Chinese Academy of Agricultural Sciences, Beijing 100193, China, [3]Institute of Agro-food Science and Technology, Chinese Academy of Agriculture Sciences, Beijing 100193, China, [4]Institute of Entomology, Jiangxi Agricultural University, Nanchang 330045, China, [5]Department of Biological Chemistry, Rothamsted Research, Harpenden, AL5 2JQ, UK.

†Correspondence and requests for materials should be addressed to Y.-J.Z. (yjzhang@ippcaas.cn) or J.-X.W. (junxw@nwsuaf.edu.cn)

*These authors contributed equally to this work.

Running title: Transcriptome and metabolome changes in cotton

Key words: cotton; *Helicoverpa armigera*, phytohormones, indirect defense, herbivore-induced volatiles, biotic stress, terpene synthase, chewing insects, transcription factor

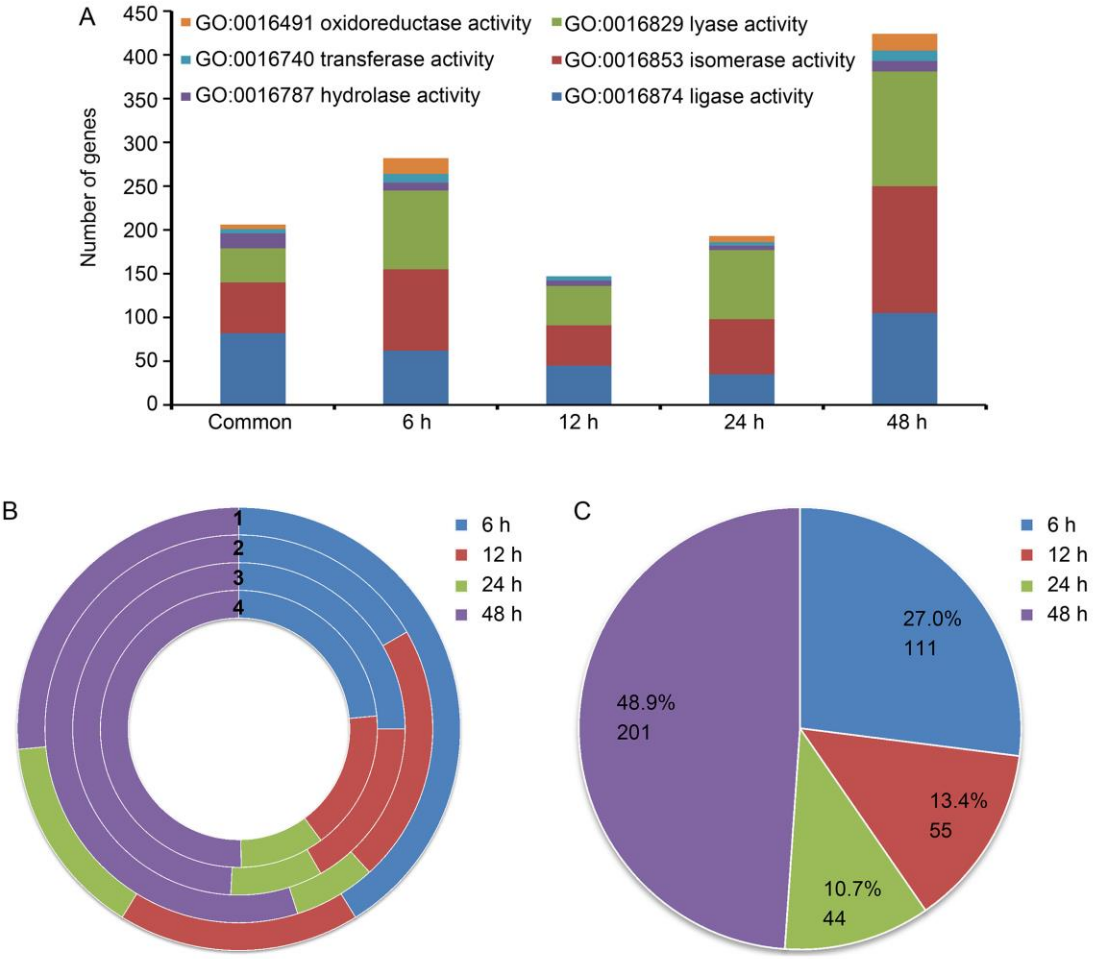

Supplementary Figure S1. GO analysis of commonly and exclusively expressed genes for the time series of 6, 12, 24 and 48h of *Helicoverpa armigera* feeding. A: GO clustering of catalytic activity in commonly and exclusively DEGs at four time points; B: GO clustering of exclusively expressed genes involved in regulation at four time points. 1, GO:0004871 signal transducer activity; 2, GO:0003700 transcription factor activity; 3, GO:0050789 regulation of biological process; 4, GO:0019222 regulation of metabolic process. C: GO clustering of exclusively expressed genes involved in biosynthetic process.

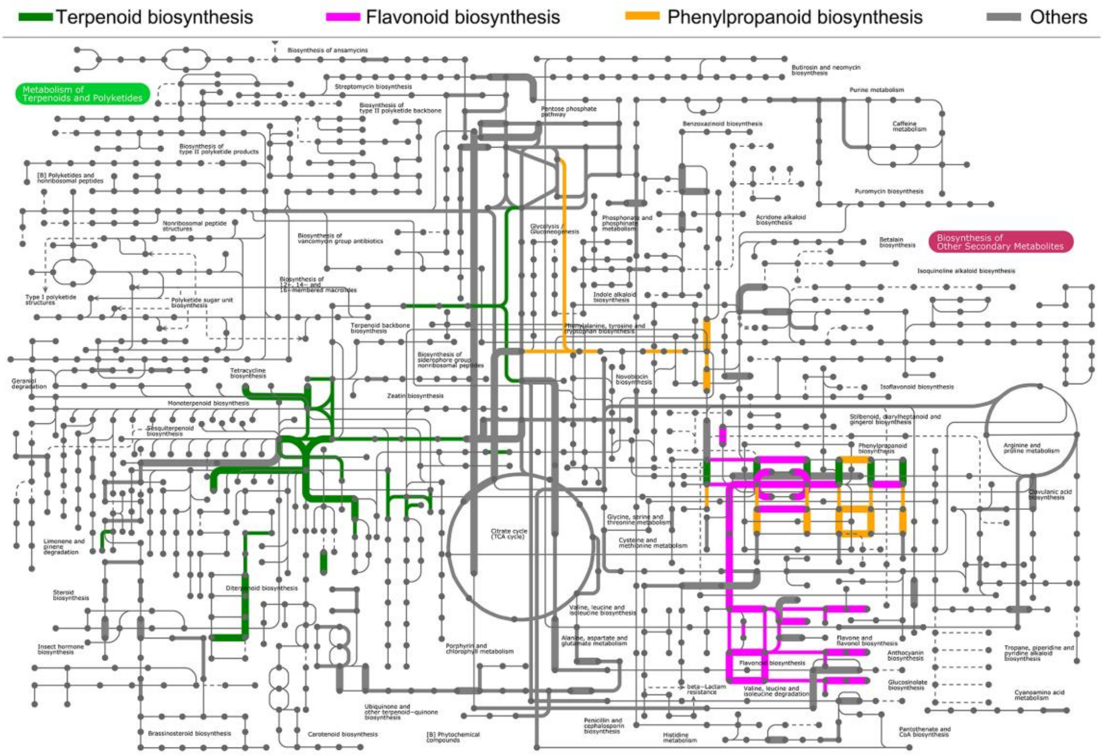

Supplementary Figure S2. JA, ET and ABA pathways expressed in *Gossypium hirsutum* after feeding by the cotton bollworm *Helicoverpa armigera*. The map was generated with iPath (http://pathways.embl.de), a web-based tool for the visualization of metabolic pathways.

Supplementary Table S2. Overall summary of differentially expressed genes at different time-points.

**Target gene**	**Type**	**6 h**	**12 h**	**24 h**	**48 h**
	Up	1422	1184	1249	2421
$q \leq 0.05$	Down	2213	1155	2367	2621
	Total	3635	2339	3616	5042
	Up	652	460	531	606
$q \leq 0.05$, $\log_2\lvert\mathrm{Ratio}\rvert \geq 2$	Down	353	207	457	319
	Total	1005	667	988	925

Supplementary Table S3. Distribution and gene ontology analysis of genes enriched in the top three clusters at the dominant response time points (6 h and 48 h).

Dominant response stage	GO annotation	Number of genes			Ratio			GO catalogue
		P1	P2	P3	P1	P2	P3	
6 h	GO:0004871 signal transducer activity	3	3	0	1.2	0.9	0	Molecular Function
	GO:0016740 transferase activity	42	23	19	16.4	6.7	5.8	Molecular Function
	GO:0016829 lyase activity	5	8	0	2	2.3	0	Molecular Function
	GO:0001882 nucleoside binding	27	22	16	10.5	6.4	4.9	Molecular Function
	GO:0009055 electron carrier activity	11	6	7	4.3	1.7	2.1	Molecular Function
	GO:0032501 multicellular organismal process	5	0	0	2	0	0	Biological Process
	GO:0031975 envelope	5	0	0	2	0	0	Cellular Component
48 h	GO:0003700 transcription factor activity	12	13	21	4.7	3.8	6.4	Molecular Function
	GO:0030528 transcription regulator activity	15	18	23	5.9	5.2	7	Molecular Function
	GO:0016787 hydrolase activity	25	35	58	9.8	10.1	17.6	Molecular Function
	GO:0016491 oxidoreductase activity	26	31	38	10.2	9	11.6	Molecular Function
	GO:0050794 regulation of cellular process	19	21	27	7.4	6.1	8.2	Biological Process
	GO:0006950 response to stress	4	4	10	1.6	1.2	3	Biological Process
	GO:0042221 response to chemical stimulus	2	5	7	0.8	1.4	2.1	Biological Process
	GO:0005576 extracellular region	0	2	10	0	0.6	3	Cellular Component

Supplementary Table S4. Overall summary of *Helicoverpa armigera*-responsive genes involved in phytohormone biosynthesis and signaling.

	Number of genes involved in phytohormone biosynthesis					Number of genes involved in phytohormone signaling				
Category	$q \leq 0.05$	$q \leq 0.05$, $\log_2$|Ratio| ≥ 2	up	down	mix	$q \leq 0.05$	$q \leq 0.05$, $\log_2$|Ratio| ≥ 2	up	down	mix
Abscisic acid	11	6	6	0	0	16	1	0	1	0
Auxin	0	0	0	0	0	55	21	11	10	0
Brassinosteroids	2	0	0	0	0	34	11	7	4	0
Cytokinin	11	3	3	0	0	40	10	5	5	0
Ethylene	18	9	8	1	0	17	6	6	0	0
Gibberellin	7	4	3	1	0	33	14	10	4	0
Jasmonate	44	26	26	0	0	25	12	12	0	0
Salicylic acid	4	4	1	3	0	6	2	0	2	0

Supplementary Table S5. Subset of *Helicoverpa armigera*-responsive genes involved in phytohormone biosynthesis and signaling. Ratio conidered significant ($q \leq 0.05$, $\log_2|\text{Ratio}| \geq 2$) are indicated in red (induced) or green (repressed).

Hormone	Probe Set ID	Maping to *Gossypium raimondii* gene	Fold Change 6h (treatment/control)	Fold Change 12h (treatment/control)	Fold Change 24h (treatment/control)	Fold Change 48h (treatment/control)
abscisic acid biosynthesis	GhiAffx.19524.1.S1_at	Gorai.007G213500.1				-0.8862435
	GhiAffx.4579.3.S1_at	Gorai.007G213500.1	-1.720188			
	Ghi.1085.2.S1_at	Gorai.002G237000.1				**2.4838733**
	GhiAffx.40958.1.S1_x_at	Gorai.002G237000.1			**2.5236929**	**2.0002569**
	Ghi.1085.1.A1_s_at	Gorai.002G237000.1			**2.4135353**	1.9432107
	Ghi.9176.4.S1_at	Gorai.002G267100.1	**2.9208064**		**2.6573368**	1.6875054
	GhiAffx.18577.1.A1_at	Gorai.002G237500.1		**3.5838197**		1.4439473
	Ghi.9176.3.A1_at	Gorai.002G267100.1	1.7919182			1.2702112
	GhiAffx.336.1.A1_at	Gorai.013G015600.1	-0.9838723		-0.5534243	-1.3985511
	GraAffx.1698.1.S1_at	Gorai.007G035400.1				-1.7850938
	Ghi.9176.3.S1_at	Gorai.002G267100.1	**2.2319123**		**2.2926252**	
abscisic acid signaling	GhiAffx.4305.1.S1_at	Gorai.006G185000.1				1.2099933
	GhiAffx.25225.1.A1_at	Gorai.004G133900.1			-1.5020979	-0.5482064
	Ghi.10676.1.S1_s_at	Gorai.007G107500.1				-0.5678034
	GhiAffx.42524.1.S1_at	Gorai.008G270200.1	-1.4970625	-1.2533122	-1.1025736	-0.9145156
	Ghi.9094.1.S1_s_at	Gorai.008G270200.1	-1.1608294	-1.3799203	-1.0923571	-1.0331427
	GraAffx.7002.1.A1_at	Gorai.008G270200.1	-1.1851737	-1.3992321	-1.3125918	-1.1768192
	Ghi.455.1.S1_at	Gorai.008G226500.1			**-2.1302775**	-1.8620519
	Ghi.9094.2.S1_at	Gorai.008G270200.1		-0.803449		
	Ghi.3008.1.S1_at	Gorai.010G076700.1			-1.0840286	-0.6176327
	Gra.489.2.A1_at	Gorai.012G072200.1	-1.1985609		-1.5786753	-0.7036874

	Gra.489.2.S1_s_at	Gorai.012G072200.1	-0.7158657		-1.45525	-1.0757475
	Ghi.3038.1.A1_s_at	Gorai.013G230300.1			-1.7632547	-1.9379481
	Ghi.10686.1.A1_at	Gorai.011G268200.1			-1.2081271	
	GhiAffx.35227.1.A1_at	Gorai.008G073200.1	-0.7716229			
	Ghi.9164.1.S1_s_at	Gorai.013G044500.1		-0.8004373	-0.8549415	
	Ghi.9296.1.S1_s_at	Gorai.009G212600.1	1.1031141			
Auxin biosynthesis						
Auxin signaling	GhiAffx.52609.1.S1_at	Gorai.010G048200.1				-1.0860297
	GhiAffx.3452.1.A1_at	Gorai.002G262100.1	-0.6832145		-0.8230264	
	Gra.1566.1.A1_at	Gorai.012G112900.1	-1.2593093	-1.575276		
	Ghi.6484.1.S1_at	Gorai.012G112900.1	-1.4373385	-1.7607562		
	GraAffx.15837.1.A1_s_at	Gorai.001G273700.1	-1.26506		-0.9745018	-0.7438908
	GraAffx.14836.1.A1_s_at	Gorai.004G085900.1	**-2.0752648**		-1.0944845	-0.7445491
	Ghi.75.1.S1_s_at	Gorai.004G140500.1	-1.3629109		-0.8444916	
	GhiAffx.15164.1.A1_s_at	Gorai.001G242800.1		**2.1459065**		**2.4909966**
	GraAffx.33168.2.A1_s_at	Gorai.004G075000.1				**2.1414248**
	Ghi.7333.1.A1_s_at	Gorai.004G222500.1		**2.4260517**		1.0198595
	Ghi.5857.1.S1_at	Gorai.009G226000.1	-1.2692195		-0.9934993	-0.4265692
	GhiAffx.34525.1.S1_s_at	Gorai.001G242900.1	-1.5618452	-1.4987606		-0.9916328
	GhiAffx.18267.1.S1_s_at	Gorai.009G135900.1			-1.791431	-1.2706884
	Ghi.8605.1.S1_s_at	Gorai.001G242900.1	-1.7195312	-1.8662672		-1.3402367
	GhiAffx.6395.1.S1_s_at	Gorai.006G246000.1	**-2.0020841**		-1.7521685	-1.606633
	Gra.2411.1.A1_s_at	Gorai.001G242900.1	-1.7679499	**-2.124986**	-1.63681	-1.668457
	Ghi.6543.1.S1_s_at	Gorai.005G046500.1	**-2.0125398**	-1.6776037	**-2.4173951**	**-2.386945**
	Ghi.2804.1.S1_s_at	Gorai.011G070800.1			-0.6713343	
	Ghi.9984.1.S1_s_at	Gorai.010G031600.1	**-2.3148644**	-1.1869403	-0.7584747	
	Ghi.10536.1.S1_s_at	Gorai.010G031600.1	**-2.2578335**		-0.8087323	
	Gra.1987.1.S1_s_at	Gorai.010G031600.1			-1.0567017	

	GhiAffx.59138.1.S1_s_at	Gorai.004G279200.1	-1.4118546		-1.2589783	
	Ghi.4821.1.S1_s_at	Gorai.009G132200.1			-1.3459612	
	Gra.1481.1.A1_s_at	Gorai.009G026700.1			-1.3630748	
	Ghi.9623.1.A1_x_at	Gorai.009G026700.1		-1.0918725	-1.4361797	
	GhiAffx.1868.1.S1_a_at	Gorai.007G150000.1	-1.0151014	-0.9221566		
	GhiAffx.62266.1.S1_at	Gorai.013G200800.1	-1.201983			
	GhiAffx.25398.1.S1_at	Gorai.009G196100.1		**3.8098119**	**2.0494927**	**3.6776534**
	Ghi.4482.1.A1_at	Gorai.002G124400.1	1.1282978	**2.5357059**	1.887176	**3.2941785**
	GhiAffx.46593.1.S1_x_at	Gorai.007G044900.1				-0.5014729
	GhiAffx.44289.1.S1_x_at	Gorai.001G054600.1				-0.5098422
	Gra.1989.1.S1_at	Gorai.007G044900.1				-0.6486977
	Ghi.5551.1.A1_s_at	Gorai.011G238900.1	-1.0961233		-1.418147	-0.7429841
	Ghi.135.1.S1_at	Gorai.006G008700.1	-1.1611913		-0.6770514	
	GhiAffx.58552.1.A1_s_at	Gorai.001G204500.1			-0.8251737	
	Ghi.5044.1.A1_s_at	Gorai.007G044900.1	-0.7432601			
	Ghi.3761.1.A1_x_at	Gorai.001G054600.1	-1.0568648			
	GarAffx.37540.1.S1_s_at	Gorai.012G009000.1	-1.1943533			
	Ghi.7615.1.S1_s_at	Gorai.001G017000.1	-1.4361985			
	GhiAffx.15786.1.S1_s_at	Gorai.005G234200.1	**4.3918343**	**5.4993478**	**4.929218**	**3.9503981**
	Ghi.4318.2.A1_s_at	Gorai.005G234200.1	**4.4481793**	**5.5134886**	**4.9681398**	**3.8861652**
	Ghi.4318.3.S1_s_at	Gorai.005G234200.1	**4.5270616**	**5.15098**	**4.0798521**	**3.4133531**
	Ghi.1324.1.S1_at	Gorai.012G030000.1		1.3136247	1.6395813	1.4564599
	Ghi.9249.4.A1_at	Gorai.007G219500.1		**4.1726446**	**3.0066732**	
	Ghi.4795.1.A1_s_at	Gorai.012G126200.1			-1.6136862	
	Ghi.4357.1.A1_x_at	Gorai.002G079600.1		**2.0088742**		
	GhiAffx.28716.1.S1_at	Gorai.004G123800.1			1.7383373	**2.0672798**
	GhiAffx.28478.1.S1_at	Gorai.004G044200.1	**-2.6401261**	-0.8844707	-1.501572	-1.4985459
	GhiAffx.3619.1.S1_at	Gorai.010G005500.1		1.6243986		-1.8223441

	GhiAffx.24789.1.S1_at	Gorai.N011800.1	**-2.7127088**		**-4.8046811**	**-4.0106464**
	GhiAffx.62188.1.S1_at	Gorai.002G124000.1	**-4.7335707**	**-3.5325928**	**-3.9645233**	**-4.0334694**
	GhiAffx.143.1.A1_at	Gorai.008G157200.1			1.4595043	
	GhiAffx.53648.1.S1_at	Gorai.002G106000.1			-1.7311337	
	GhiAffx.22909.1.S1_at	Gorai.008G032600.1			**-4.5604252**	
	GhiAffx.25154.1.A1_at	Gorai.009G144300.1	-1.5141855			
brassinosteroids biosynthesis	Ghi.1119.1.S1_s_at	Gorai.002G062200.1			-1.1097237	
	Ghi.917.1.S1_at	Gorai.012G111500.1	-1.0017757			
brassinosteroids signaling	Ghi.1440.3.S1_at	Gorai.011G052300.1		**2.2126059**	**2.2020434**	**2.5757515**
	GhiAffx.47936.1.S1_at	Gorai.008G006500.1	**2.8253486**		**2.524109**	**2.008052**
	Ghi.5628.2.S1_s_at	Gorai.009G025100.1		1.2527535	1.2583882	1.5359714
	GhiAffx.40483.1.S1_at	Gorai.001G002500.1			1.7390019	1.5096999
	GraAffx.26883.1.A1_s_at	Gorai.011G052300.1			1.3433963	1.4995199
	Ghi.3567.2.S1_at	Gorai.002G049000.1				0.8466042
	Ghi.3774.1.S1_at	Gorai.011G052000.1				0.8220781
	GhiAffx.21794.1.A1_at	Gorai.009G127800.1				0.4117752
	Ghi.3915.1.A1_x_at	Gorai.005G138000.1				-0.555116
	GhiAffx.24962.1.A1_s_at	Gorai.008G020900.1	**2.3995763**		1.1234691	
	Gra.2451.1.S1_x_at	Gorai.009G105500.1			0.7105808	
	Ghi.3820.1.S1_at	Gorai.003G153500.1	-1.4581231	-1.4801089	-1.4229772	
	Ghi.1573.1.S1_at	Gorai.008G086100.1	**-2.5715321**		**-2.1446134**	
	GhiAffx.12960.1.A1_at	Gorai.005G075700.1	-1.2514924	-1.4823484		
	Ghi.9278.2.S1_s_at	Gorai.008G020900.1	**2.268034**			
	GhiAffx.24962.1.S1_x_at	Gorai.008G020900.1	**2.1551866**			
	Ghi.8887.1.S1_s_at	Gorai.007G186000.1	-1.0770843			-0.5731511
	Ghi.5708.1.A1_at	Gorai.012G058000.1			-0.7741853	-0.5770373
	GhiAffx.24518.1.S1_s_at	Gorai.003G048200.1	**-2.2643237**	-1.5140823	-1.9832718	-0.8846206
	Ghi.3937.1.A1_x_at	Gorai.003G048200.1	-1.6065437	-1.2195965	-1.1331882	-0.9323307

	GhiAffx.15808.1.A1_at	Gorai.010G219700.1	**-2.0146279**			-1.6419226
	GhiAffx.2573.1.A1_at	Gorai.011G001200.1			1.3262928	
	Ghi.1831.1.S1_s_at	Gorai.003G082600.1			-1.0229866	
	Ghi.4900.1.A1_x_at	Gorai.005G188100.1	**-2.5815019**		-1.6660572	
	Ghi.124.1.S1_s_at	Gorai.009G188600.1		1.8436821		**2.117931**
	Ghi.1640.1.S1_at	Gorai.006G179500.1				1.1924867
	Ghi.8818.1.S1_at	Gorai.009G094800.1			-1.5333774	-1.0433436
	GhiAffx.43685.1.S1_s_at	Gorai.005G113100.1			-0.4352928	
	Ghi.2737.1.S1_at	Gorai.006G089300.1				0.6290964
	Ghi.8007.1.S1_at	Gorai.011G036300.1	1.7764329	**2.6721125**	**2.2098243**	**2.7290328**
	Ghi.4715.1.A1_at	Gorai.002G052400.1				0.5293139
	GhiAffx.40750.1.A1_s_at	Gorai.009G274000.1				-0.5557798
	GhiAffx.23450.1.A1_s_at	Gorai.010G240700.1			-0.9378579	
	GraAffx.2170.1.S1_s_at	Gorai.008G078400.1			-1.3798139	-1.3692622
cytokinin biosynthesis	Ghi.5389.1.A1_at	Gorai.005G032800.1		**3.0682522**		**3.6214536**
	Ghi.3637.1.A1_at	Gorai.005G033200.1		**2.1100859**	**2.1375184**	**2.1665092**
	Ghi.3620.1.S1_x_at	Gorai.013G041300.1			1.2263831	0.9549116
	Ghi.7673.1.A1_s_at	Gorai.011G158000.1				0.8937557
	Gra.1171.1.A1_s_at	Gorai.011G158000.1				0.8879821
	Gra.1732.4.S1_at	Gorai.013G041300.1			0.8556903	0.7686076
	Ghi.7673.1.S1_s_at	Gorai.011G158000.1				0.7150351
	Gra.1732.1.A1_s_at	Gorai.013G041300.1			0.755541	0.6641615
	Ghi.190.1.S1_at	Gorai.001G113200.1	**2.5226124**		**2.6947542**	
	Ghi.5875.1.A1_at	Gorai.009G066700.1			-1.1902652	
	GhiAffx.25127.1.A1_at	Gorai.001G208700.1		-1.7437437		
cytokinin signaling	GhiAffx.31991.1.A1_s_at	Gorai.009G442700.1		**2.3310553**		1.8507104
	GhiAffx.63687.1.S1_at	Gorai.004G094500.1				0.8588788
	Ghi.5742.1.A1_s_at	Gorai.008G177700.1				0.6283644

	Ghi.7551.1.S1_at	Gorai.003G101000.1			**-2.7358767**	**-2.3076934**
	GraAffx.15515.1.A1_x_at	Gorai.005G243100.1	**2.0959904**		1.2784625	
	Gra.1576.1.A1_s_at	Gorai.009G179400.1			-1.0405529	
	GhiAffx.24601.1.A1_a_at	Gorai.010G078300.1	-1.7319137		-1.4405439	
	Ghi.8881.1.S1_at	Gorai.001G204900.1			-1.5057026	
	Ghi.7518.3.S1_x_at	Gorai.013G093600.1			-1.7021767	
	Ghi.7518.1.S1_s_at	Gorai.013G093600.1			-1.7913255	
	GhiAffx.53777.1.S1_s_at	Gorai.001G216900.1			**-2.829245**	
	Ghi.9269.1.S1_at	Gorai.013G263800.1		**3.5303877**		
	Ghi.2113.1.S1_at	Gorai.013G031500.1		1.394405		
	GhiAffx.8593.1.A1_s_at	Gorai.008G170200.1		-0.905756		
	GhiAffx.8593.1.S1_s_at	Gorai.008G170200.1		-1.8095071		
	Ghi.9269.2.S1_s_at	Gorai.013G263800.1	**3.1634769**			
	GhiAffx.32625.1.S1_s_at	Gorai.001G204900.1	1.180217			
	Ghi.7011.1.A1_s_at	Gorai.007G291700.1				0.9472844
	Gra.511.1.A1_s_at	Gorai.011G182900.1				-0.4660916
	Ghi.7529.1.A1_s_at	Gorai.011G182900.1			-0.6569661	-0.5629959
	Ghi.7529.1.S1_s_at	Gorai.011G182900.1			-0.4118015	
	Ghi.3434.1.S1_at	Gorai.008G006900.1	-0.7660072			
	Ghi.501.2.S1_at	Gorai.008G185500.1		1.4519521	**2.1696839**	**2.105351**
	Ghi.501.2.A1_at	Gorai.008G185500.1			1.774028	1.8640738
	GhiAffx.39948.1.S1_at	Gorai.008G073300.1		1.344777	1.787997	1.8090606
	GhiAffx.59955.1.S1_at	Gorai.003G021300.1		1.2993638	1.8086966	1.6182096
	Ghi.3627.1.A1_at	Gorai.001G030600.1		1.8691104	1.5332675	0.4708601
	GhiAffx.62834.1.S1_at	Gorai.003G180100.1				-0.8259846
	GhiAffx.3456.1.A1_a_at	Gorai.009G120900.1	-1.4903387	**-2.0732857**	-1.8439485	-0.9893128
	GraAffx.1960.1.S1_at	Gorai.007G287500.1				-1.1187611
	GhiAffx.47163.1.S1_at	Gorai.005G173300.1			-1.8202582	-1.1993153

	GhiAffx.40609.1.S1_at	Gorai.005G033900.1			-1.5737235	
	Ghi.3627.1.S1_at	Gorai.001G030600.1		1.3961778		
	Gra.1734.1.A1_s_at	Gorai.006G222400.1		-0.8422906		
	Ghi.5072.1.A1_s_at	Gorai.012G150500.1	**-2.4891572**	-1.028398		
	GhiAffx.6545.1.S1_at	Gorai.008G155000.1		-1.640582		
	Ghi.6741.1.S1_at	Gorai.008G280500.1	1.3981761			
	GraAffx.4152.1.S1_at	Gorai.010G051300.1	-0.9610682			
	Ghi.8924.2.A1_s_at	Gorai.010G051300.1	-1.9065481			
	Ghi.8924.1.S1_s_at	Gorai.010G051300.1	**-2.1567437**			
ethylene biosynthesis	Ghi.1014.1.S1_s_at	Gorai.004G146800.1	0.8561571		0.9600232	0.9666046
	Gra.1668.1.A1_s_at	Gorai.012G116000.1			-0.627212	-0.7046579
	GraAffx.33524.1.S1_s_at	Gorai.012G116000.1			-0.9711812	-1.0623686
	Ghi.7628.1.S1_at	Gorai.005G211000.1		-0.569997	-0.8504678	
	Ghi.10003.1.S1_s_at	Gorai.012G134300.1		-1.5662709	**-2.23656**	
	Ghi.9616.1.S1_s_at	Gorai.005G071300.1	**3.6548237**	**2.8248833**		
	Gra.2009.1.S1_s_at	Gorai.005G071300.1	**2.9586703**			
	Ghi.5451.1.S1_at	Gorai.008G250100.1	**4.4446108**	**3.4721458**	**3.3246366**	**3.3359513**
	Ghi.23.4.A1_at	Gorai.007G103200.1	**4.8264718**	**3.780676**		**2.4783095**
	Gra.2961.1.S1_a_at	Gorai.004G062100.1	1.3627166			0.9207155
	Ghi.798.1.S1_s_at	Gorai.004G062100.1	0.7814742		0.7880792	0.7094569
	Ghi.7921.1.S1_x_at	Gorai.001G096300.1	**3.6209247**		**3.4748095**	
	Ghi.8025.1.S1_s_at	Gorai.001G096300.1	**4.2648072**		1.7857877	
	Gra.2961.2.A1_x_at	Gorai.004G062100.1			1.3433811	
	GhiAffx.60763.1.S1_at	Gorai.001G072100.1			-0.6573929	
	GraAffx.20159.1.A1_s_at	Gorai.007G170100.1		**3.313951**		
	Gra.647.1.A1_s_at	Gorai.001G096300.1	**2.8298515**			
	Ghi.6953.1.S1_s_at	Gorai.009G182300.1	1.5727253			
ethylene signaling	GhiAffx.31527.1.S1_s_at	Gorai.008G143100.1	1.2315583		**2.0705427**	-0.7020485

	Ghi.3204.1.S1_at	Gorai.005G047900.1			**2.2424183**	
	Ghi.5775.1.S1_s_at	Gorai.006G247800.1		1.7178783	1.9789894	
	Gra.1441.1.A1_at	Gorai.005G047900.1			1.4057223	
	Ghi.5775.1.A1_at	Gorai.006G247800.1		1.2881356	1.3003862	
	Ghi.6993.1.A1_at	Gorai.005G004900.1		-0.8073722	-0.8506272	-0.8226277
	GhiAffx.16540.2.S1_at	Gorai.007G076700.1	-1.3675711		-1.2415881	
	Ghi.757.1.S1_x_at	Gorai.005G123100.1		0.5540987		
	GhiAffx.16184.1.S1_at	Gorai.004G227800.1	1.3292196	1.2801857	1.9075882	1.450078
	Ghi.7115.1.S1_s_at	Gorai.004G227800.1				0.7048017
	Gra.260.2.S1_s_at	Gorai.004G227800.1			0.8221301	0.5554792
	GhiAffx.43606.1.S1_s_at	Gorai.013G266300.1		0.5273018		
	Ghi.4158.1.A1_s_at	Gorai.009G058000.1				-1.2093961
	GhiAffx.31721.2.S1_s_at	Gorai.009G017100.1		1.8154231	**2.6301045**	
	Gra.1585.1.A1_at	Gorai.009G017100.1		1.6520902	**2.3013617**	
	GhiAffx.24637.1.S1_at	Gorai.011G173000.1			**4.5583477**	
	GhiAffx.39399.1.S1_at	Gorai.010G156600.1		**2.4812047**	**2.0288213**	1.5542675
gibberellin biosynthesis	GhiAffx.13394.2.S1_x_at	Gorai.004G187100.1		-1.394983		
	GhiAffx.12577.1.S1_at	Gorai.001G047500.1				1.4117924
	GraAffx.15730.1.S1_at	Gorai.006G005500.1	**-3.3160196**		-1.626301	-0.6493604
	Ghi.9280.3.A1_at	Gorai.013G028600.1		**2.2074117**		
	Ghi.8028.1.S1_a_at	Gorai.001G173000.1	-1.6491532			
	GhiAffx.4691.1.A1_at	Gorai.002G038900.1	**4.3621167**			
	Ghi.1172.3.A1_at	Gorai.001G212500.1	1.3557291	**3.6258788**	**2.3219674**	**2.8745244**
gibberellin signaling	Ghi.10228.1.S1_at	Gorai.007G132800.1		**2.2856217**	**3.0380148**	**3.0702667**
	Gra.1544.1.A1_s_at	Gorai.008G007200.1		1.5935449	**2.6142024**	**2.4271406**
	Gra.1079.1.A1_s_at	Gorai.011G116900.1				1.6660769
	Gra.2150.1.S1_s_at	Gorai.010G215600.1	**2.2367032**		**2.2021389**	1.4606262
	GhiAffx.43317.1.A1_s_at	Gorai.004G244100.1		1.7856111		0.9717157

	GhiAffx.24606.1.S1_a_at	Gorai.005G239100.1	-1.8068965		-1.0594284	-0.7438932
	Gra.49.1.S1_at	Gorai.007G093200.1			-1.3543684	-1.0323157
	Ghi.10345.1.S1_at	Gorai.005G239400.1		-1.3219264		-1.2843445
	Ghi.6309.2.A1_at	Gorai.003G140500.1	-1.0447101		**-2.2088823**	-1.6137192
	GraAffx.27014.1.S1_at	Gorai.008G282100.1			**2.3764879**	
	Gra.2150.1.A1_s_at	Gorai.010G215600.1	1.2204381		0.902183	
	Ghi.10329.1.S1_at	Gorai.009G427800.1	**3.7795897**	**2.9642662**	**3.3634345**	**3.5826968**
	Ghi.3132.1.S1_at	Gorai.010G249400.1				**2.6603318**
	Ghi.9123.1.S1_at	Gorai.003G130400.1				1.5178408
	Ghi.3126.1.S1_s_at	Gorai.002G096300.1	**2.2746261**	1.9718972		1.4791111
	GhiAffx.44794.1.S1_at	Gorai.009G098100.1	**2.0003416**		**3.2320932**	1.4236697
	Ghi.9302.1.A1_at	Gorai.005G083700.1	1.3151462		1.2998532	1.2443473
	GhiAffx.63632.1.S1_at	Gorai.006G198300.1		1.1278661		1.1711208
	Ghi.4703.1.A1_s_at	Gorai.002G096300.1	1.6639585	1.382271		0.9636425
	Ghi.4006.1.S1_s_at	Gorai.001G089400.1	-1.6154262		-1.0545278	-0.8075173
	Ghi.9219.2.A1_s_at	Gorai.007G167000.1	**-2.0476641**		-1.5406668	-0.8597906
	GhiAffx.16118.1.S1_s_at	Gorai.001G089400.1	-1.82952		-1.1261219	-0.8887603
	GhiAffx.34665.1.S1_at	Gorai.009G021700.1			**-4.4072687**	**-2.6578812**
	Ghi.9360.1.S1_at	Gorai.002G080600.1	**3.4842843**	**2.3743562**		
	Gra.1358.1.A1_at	Gorai.013G164700.1	**3.5355274**			
	Ghi.9360.1.A1_at	Gorai.002G080600.1	1.4494045			
	GraAffx.21485.1.A1_s_at	Gorai.002G096300.1	1.3524019			
	GhiAffx.44405.1.A1_at	Gorai.003G130600.1	-1.4060403			
	GhiAffx.3767.1.S1_at	Gorai.011G266200.1	-1.6986295			
	Ghi.4703.1.S1_at	Gorai.002G096300.1	1.4955818	1.1950597		0.9421741
	GhiAffx.23338.1.S1_at	Gorai.008G068100.1	**-2.5578997**			
	GhiAffx.7427.1.S1_s_at	Gorai.010G023800.1				-0.6565977
	Ghi.216.1.S1_at	Gorai.009G276600.1			-1.8107831	

jasmonic acid biosynthesis	GhiAffx.1589.46.S1_s_at	Gorai.011G060400.1	**5.1891176**	**2.8958415**	**3.4234277**	**4.0514131**
	Ghi.1739.3.S1_s_at	Gorai.011G060400.1	**4.3588082**	**3.0089016**	**3.5093504**	**3.9679183**
	Ghi.1739.2.S1_s_at	Gorai.011G060400.1	**4.387215**	**2.5810415**	**3.1187925**	**3.459614**
	Ghi.1739.4.S1_x_at	Gorai.011G060400.1	**4.326164**	**2.4232644**	**3.168018**	**3.4307845**
	Gra.2818.1.S1_s_at	Gorai.011G060400.1	**3.3574457**	**2.5720634**	**2.3165107**	**2.4846599**
	Gra.2995.1.S1_s_at	Gorai.009G071200.1	1.9291246	**2.5395642**	1.8679508	1.0406001
	Ghi.234.1.S1_s_at	Gorai.013G124500.1	1.4485501	1.9037224	**2.2539166**	0.7789183
	Gra.38.1.A1_at	Gorai.009G071200.1		0.8729717		-0.5170985
	GraAffx.18873.1.A1_s_at	Gorai.009G071200.1		1.61268	1.2252208	
	Gra.38.1.S1_s_at	Gorai.009G071200.1		1.9249466		
	GraAffx.17938.1.S1_at	Gorai.013G124500.1	1.5245376			
	Ghi.5304.1.A1_at	Gorai.010G013600.1			**4.0406219**	**4.8582626**
	Ghi.10001.1.A1_at	Gorai.009G274700.1	**4.0978258**	**4.3170024**	**3.0967019**	**3.8538753**
	Ghi.10001.2.S1_at	Gorai.009G274700.1	**4.26274**	**4.2736486**	**2.8607885**	**3.295087**
	Ghi.10001.1.S1_s_at	Gorai.009G274700.1	**3.5553211**	**3.3018549**	**2.2415768**	**2.8342986**
	GhiAffx.47143.1.S1_at	Gorai.008G044100.1		**2.6994503**	1.6653422	
	Gra.1457.1.S1_at	Gorai.010G013700.1		**3.5331187**		
	Ghi.5304.2.A1_x_at	Gorai.010G013600.1		**2.529524**		
	Ghi.2998.1.A1_x_at	Gorai.004G046300.1	**2.9236082**	**3.7565927**	**2.3395875**	**2.7846758**
	Ghi.2998.2.S1_x_at	Gorai.004G046100.1	**2.8291459**	**3.2615654**	**2.1382046**	**2.2297563**
	Ghi.1032.3.S1_at	Gorai.009G035400.1	**2.9661876**	**2.6801474**	**2.3543023**	**2.1644411**
	Ghi.1032.2.S1_x_at	Gorai.009G035400.1	**2.1871497**	**2.2733648**	**2.2644874**	**2.0940419**
	Ghi.1032.2.A1_s_at	Gorai.009G035400.1	1.9983465	**2.0410896**	**2.0473945**	1.9085915
	GraAffx.28706.1.S1_s_at	Gorai.009G035400.1	**2.7516782**			
	Ghi.9852.2.S1_x_at	Gorai.005G113000.1	1.8484906	0.6485184	1.2695339	0.9898394
	Ghi.9852.1.S1_a_at	Gorai.005G113000.1	1.659823		1.1437745	
	GhiAffx.9578.1.S1_at	Gorai.007G240800.1		1.6565552		1.4423068
	Ghi.6402.1.S1_at	Gorai.007G240800.1		1.6717971	1.1091169	1.3403029

	GhiAffx.55382.1.S1_at	Gorai.007G240800.1		1.3488228	0.8929315	1.2948565
	Gra.2973.1.S1_s_at	Gorai.008G233600.1	1.8810126	**2.0888581**	1.0124414	1.1810373
	Ghi.8480.1.S1_s_at	Gorai.002G020600.1				0.7927177
	Ghi.8572.1.S1_at	Gorai.002G020600.1				0.7915252
	Ghi.7466.1.S1_at	Gorai.001G247400.1		1.021813		0.5782586
	GhiAffx.22064.1.S1_at	Gorai.002G043200.1			1.1622051	
	Ghi.8582.1.A1_at	Gorai.004G280500.1		0.7992292		0.4566473
	Gra.1505.1.S1_s_at	Gorai.006G076600.1		0.9167254		
	GhiAffx.5185.1.S1_s_at	Gorai.011G191800.1	-0.6059848			
	Gra.1971.1.A1_at	Gorai.006G261300.1		**2.6606427**	1.7404781	**2.1675892**
	GhiAffx.40066.1.A1_at	Gorai.001G185200.1	**2.6492525**	**3.054829**		1.6074675
	Ghi.10732.1.S1_s_at	Gorai.011G161700.1		0.7345979		1.0249805
	Ghi.10704.1.S1_s_at	Gorai.007G152600.1	1.1490331	1.0598746		0.6683675
	Gra.1971.1.S1_s_at	Gorai.006G261300.1		**2.5408969**		
	GhiAffx.43038.1.S1_at	Gorai.008G065900.1	**3.353807**	**4.2442809**	**2.3661186**	**2.3843032**
	GraAffx.11740.1.A1_s_at	Gorai.008G065900.1	**2.1416476**	**4.5622752**		
jasmonic acid signaling	Ghi.791.1.S1_s_at	Gorai.011G279900.1	-1.3410053	-0.7283814		
	GhiAffx.14120.1.A1_at	Gorai.011G279900.1		-0.7549179		
	Ghi.3282.1.A1_x_at	Gorai.010G090600.1	**4.6792055**	**4.2921235**	**4.3895697**	**4.5451322**
	Ghi.10327.1.S1_s_at	Gorai.004G285100.1	**4.1154684**	**3.8135515**	**3.7306726**	**3.756644**
	Ghi.9871.1.S1_at	Gorai.009G036800.1	**2.9070051**	**3.9923369**	**3.1820238**	**3.2075071**
	Ghi.269.1.S1_at	Gorai.011G062000.1	**2.4285165**	**2.3793427**	**2.1386377**	**2.2227318**
	GhiAffx.42068.1.S1_at	Gorai.002G173700.1	**2.7996147**	**2.0589596**	1.8456009	1.9532114
	Gra.2764.1.A1_s_at	Gorai.002G173700.1	1.9219247	**2.537106**	1.5787565	1.0253096
	GraAffx.26810.2.S1_s_at	Gorai.009G039500.1		-0.5015006	-0.6231148	-0.395362
	Ghi.6077.1.S1_at	Gorai.001G018800.1	-0.6659847		-0.6477185	-0.5453139
	GraAffx.26810.1.A1_at	Gorai.009G039500.1			-0.752761	-0.609963
	GraAffx.8846.1.S1_at	Gorai.001G018800.1	-0.7360109		-0.5928738	-0.7200465

	Ghi.1706.1.S1_s_at	Gorai.009G330500.1			-0.7409713	
	Gra.1625.1.A1_s_at	Gorai.009G036800.1	**4.1035577**	**3.8699005**		
	Gra.1831.1.A1_s_at	Gorai.009G145400.1		**2.8127335**		
	GhiAffx.60504.1.A1_s_at	Gorai.007G229000.1	**4.248856**	**5.0068557**	**3.0799639**	**4.0707348**
	GhiAffx.7737.1.S1_at	Gorai.007G352300.1		**2.0232186**	1.9607345	**2.2829613**
	Ghi.5471.1.S1_at	Gorai.004G184800.1	1.9296146	1.6150898	1.1239642	1.7502628
	GhiAffx.33252.1.S1_at	Gorai.003G035900.1				1.2031517
	Ghi.8777.1.S1_at	Gorai.007G109300.1	1.1113965	1.878605	1.0321223	1.1984099
	GhiAffx.10340.1.S1_at	Gorai.013G242800.1	1.474392			1.011556
	GraAffx.15333.1.A1_s_at	Gorai.004G212200.1				-1.0558537
	GraAffx.23349.1.S1_s_at	Gorai.008G259000.1				-1.2159146
	GhiAffx.44107.1.S1_at	Gorai.004G149500.1		**3.6160095**		
	Ghi.7949.1.S1_s_at	Gorai.007G023500.1		**2.608573**		
salicylic acid biosynthesis	Ghi.4039.1.A1_s_at	Gorai.002G248000.1				**-2.2438331**
	Gra.2239.1.S1_s_at	Gorai.002G248000.1				**-2.7037683**
	Gra.2682.2.S1_s_at	Gorai.002G248000.1				**-3.2065204**
	Ghi.3957.2.S1_at	Gorai.009G416300.1	**2.9105658**			
salicylic acid signaling	Ghi.3296.1.S1_at	Gorai.011G050200.1				1.0776524
	Ghi.3296.1.A1_at	Gorai.011G050200.1				1.0364033
	Gra.1569.1.A1_at	Gorai.011G217200.1			-0.4654827	-0.5117897
	Ghi.6433.1.S1_at	Gorai.008G044000.1				1.159175
	GhiAffx.21879.1.S1_at	Gorai.004G101200.1			**-4.1484605**	**-3.168335**
	GhiAffx.60328.1.S1_at	Gorai.004G101300.1			**-3.2549733**	**-3.7156531**

Supplementary Table S6. *Helicoverpa armigera*-responsive transcription factors. Ratio conidered significant ($q \leq 0.05$, $\log_2|\text{Ratio}| \geq 2$) are indicated in red (induced) or green (repressed).

Probe Set ID	Maping to *Gossypium raimondii* gene	TF family	Fold Change 6h (treatment/control)	Fold Change 12h (treatment/control)	Fold Change 24h (treatment/control)	Fold Change 48h (treatment/control)
Gra.2948.4.A1_s_at	Gorai.003G139800.1	AP2	-1.298602365		-1.09857031	-0.650619825
Gra.2948.2.S1_at	Gorai.003G139800.1	AP2	-1.42320279		-1.502486187	
GhiAffx.50950.1.A1_at	Gorai.013G003400.1	AP2		**4.912198629**		
Ghi.4744.1.A1_at	Gorai.003G091500.1	AP2	-0.682520148			
GhiAffx.25398.1.S1_at	Gorai.009G196100.1	ARF		**3.809811887**	**2.049492655**	**3.677653351**
Ghi.4482.1.A1_at	Gorai.002G124400.1	ARF	1.128297844	**2.535705906**	1.887176017	**3.294178513**
GhiAffx.46593.1.S1_x_at	Gorai.007G044900.1	ARF				-0.501472902
GhiAffx.44289.1.S1_x_at	Gorai.001G054600.1	ARF				-0.509842165
Gra.1989.1.S1_at	Gorai.007G044900.1	ARF				-0.648697702
Ghi.5551.1.A1_s_at	Gorai.011G238900.1	ARF	-1.096123259		-1.418146978	-0.74298405
Ghi.135.1.S1_at	Gorai.006G008700.1	ARF	-1.161191279		-0.677051368	
GhiAffx.58552.1.A1_s_at	Gorai.001G204500.1	ARF			-0.825173666	
Ghi.5044.1.A1_s_at	Gorai.007G044900.1	ARF	-0.74326009			
Ghi.3761.1.A1_x_at	Gorai.001G054600.1	ARF	-1.056864767			
GarAffx.37540.1.S1_s_at	Gorai.012G009000.1	ARF	-1.194353289			
Ghi.7615.1.S1_s_at	Gorai.001G017000.1	ARF	-1.436198549			
Ghi.3627.1.A1_at	Gorai.001G030600.1	ARR-B		1.869110375	1.533267492	0.470860087
GhiAffx.62834.1.S1_at	Gorai.003G180100.1	ARR-B				-0.825984631
GhiAffx.47163.1.S1_at	Gorai.005G173300.1	ARR-B			-1.820258222	-1.199315252
Ghi.3627.1.S1_at	Gorai.001G030600.1	ARR-B		1.396177838		
Ghi.6741.1.S1_at	Gorai.008G280500.1	ARR-B	1.398176136			
GhiAffx.42355.1.A1_at	Gorai.002G061900.1	B3			**2.593188294**	**3.008442685**
Ghi.5626.1.S1_s_at	Gorai.011G111100.1	B3			1.432452791	1.452347941

GhiAffx.15554.1.S1_x_at	Gorai.013G039800.1	B3				-0.326413653
GhiAffx.40544.1.S1_at	Gorai.007G114200.1	B3	-1.065773595			-0.390079891
GhiAffx.49300.1.S1_s_at	Gorai.002G130300.1	B3	-1.563426207		-0.997428174	-0.430958869
GhiAffx.25323.1.S1_at	Gorai.005G105400.1	B3	-1.039900884		-0.854408787	-0.700366418
GhiAffx.8390.1.S1_at	Gorai.012G150900.1	B3			-1.468485058	
GhiAffx.15554.1.A1_s_at	Gorai.013G039800.1	B3	-0.540769083			
GhiAffx.31804.1.S1_at	Gorai.002G131600.1	B3	-1.021187305			
Ghi.3735.1.A1_at	Gorai.008G071300.1	B3	-1.128136593			
Ghi.8007.1.S1_at	Gorai.011G036300.1	BES1	1.77643292	**2.672112525**	**2.209824283**	**2.729032833**
Ghi.4715.1.A1_at	Gorai.002G052400.1	BES1				0.529313882
GhiAffx.40750.1.A1_s_at	Gorai.009G274000.1	BES1				-0.555779821
GhiAffx.23450.1.A1_s_at	Gorai.010G240700.1	BES1			-0.937857882	
GhiAffx.60504.1.A1_s_at	Gorai.007G229000.1	bHLH	**4.248855988**	**5.006855694**	**3.0799639**	**4.070734833**
GhiAffx.7737.1.S1_at	Gorai.007G352300.1	bHLH		**2.023218573**	1.960734508	**2.282961264**
GhiAffx.28787.1.S1_at	Gorai.009G002200.1	bHLH	**4.007633421**	**2.441518924**	**2.618321098**	1.898457397
Ghi.1312.1.S1_s_at	Gorai.007G044400.1	bHLH				1.768565899
Ghi.5471.1.S1_at	Gorai.004G184800.1	bHLH	1.929614576	1.615089818	1.12396416	1.750262812
Ghi.9282.1.S1_s_at	Gorai.004G035800.1	bHLH	**2.124735825**	1.518506688	1.304329543	1.65925618
GhiAffx.28787.1.A1_at	Gorai.009G002200.1	bHLH	**3.157222446**	1.939184366	1.644927182	1.314414041
Ghi.1743.1.S1_at	Gorai.010G112200.1	bHLH			1.029372087	1.215515772
Ghi.4315.1.S1_at	Gorai.005G012400.1	bHLH				1.204210813
GhiAffx.33252.1.S1_at	Gorai.003G035900.1	bHLH				1.203151662
Ghi.8777.1.S1_at	Gorai.007G109300.1	bHLH	1.111396468	1.878605038	1.032122254	1.198409923
Ghi.6776.1.S1_at	Gorai.011G020100.1	bHLH	**2.759563455**		1.446612555	1.014565207
GhiAffx.10340.1.S1_at	Gorai.013G242800.1	bHLH	1.474392008			1.011555958
Ghi.792.1.S1_x_at	Gorai.007G314800.1	bHLH				0.669320818
Ghi.8125.1.S1_x_at	Gorai.007G034400.1	bHLH				0.528469697
GhiAffx.44161.1.S1_s_at	Gorai.011G073400.1	bHLH		-1.187936673		-0.746697886
GhiAffx.60714.1.S1_at	Gorai.002G081900.1	bHLH				-0.840127723

GraAffx.15333.1.A1_s_at	Gorai.004G212200.1	bHLH				-1.055853679
GhiAffx.25241.1.S1_x_at	Gorai.009G176000.1	bHLH	-1.338337829	-1.381824182	-1.855474905	-1.186005965
GraAffx.23349.1.S1_s_at	Gorai.008G259000.1	bHLH				-1.21591459
Ghi.6289.1.S1_at	Gorai.010G028200.1	bHLH				-1.256668991
Ghi.4223.1.S1_s_at	Gorai.003G099500.1	bHLH	**-3.061808644**	**-2.043851142**	**-2.36372728**	-1.518399001
Ghi.4223.1.A1_s_at	Gorai.003G099500.1	bHLH	**-2.34855298**		**-2.489793375**	-1.929914274
GhiAffx.61724.1.S1_at	Gorai.007G294800.1	bHLH				**-2.733478305**
GhiAffx.7727.1.A1_at	Gorai.008G209900.1	bHLH			**-3.28795609**	**-2.971747398**
GhiAffx.62126.1.S1_a_at	Gorai.008G154300.1	bHLH			-1.101651812	
GraAffx.16018.2.A1_a_at	Gorai.002G229100.1	bHLH	-1.28320948		-1.300769641	
Ghi.1543.2.S1_at	Gorai.007G361700.1	bHLH	-1.466639139		-1.31343881	
Ghi.9180.2.S1_s_at	Gorai.005G136000.1	bHLH	**3.565522363**		-1.840575329	
GhiAffx.44107.1.S1_at	Gorai.004G149500.1	bHLH		**3.616009486**		
Ghi.7949.1.S1_s_at	Gorai.007G023500.1	bHLH		**2.608573024**		
Ghi.9470.1.A1_at	Gorai.007G141800.1	bHLH	-0.615207212	0.778570868		
GhiAffx.46536.1.A1_at	Gorai.008G175100.1	bHLH	-1.368820486	-0.652029712		
Ghi.9180.2.A1_s_at	Gorai.005G136000.1	bHLH	**2.761882303**			
GraAffx.26585.1.S1_at	Gorai.013G138300.1	bHLH	-0.543901005			
Ghi.7028.1.S1_at	Gorai.013G098000.1	bHLH	-0.810360114			
GhiAffx.26756.1.S1_s_at	Gorai.008G106900.1	bHLH	-1.588502585			
Gra.35.1.A1_s_at	Gorai.008G106900.1	bHLH	-1.797012084			
Ghi.10259.1.S1_s_at	Gorai.001G144200.1	bZIP	**2.148659424**	**2.064686699**	1.606996396	**2.088044562**
GraAffx.16064.1.A1_s_at	Gorai.001G144200.1	bZIP	**2.174813035**	1.957579454	1.415640981	1.751500268
GraAffx.553.1.A1_at	Gorai.012G007100.1	bZIP		1.172544946		1.404190294
GraAffx.31296.1.S1_at	Gorai.004G153100.1	bZIP	1.519968834			1.182205455
Ghi.4643.1.A1_s_at	Gorai.009G199300.1	bZIP		1.250094868		1.098789584
Ghi.3805.1.S1_at	Gorai.007G277300.1	bZIP		**2.833127198**		0.839860471
Ghi.3241.3.S1_s_at	Gorai.004G153100.1	bZIP				0.733445038
Ghi.2271.1.S1_at	Gorai.007G205700.1	bZIP				0.721901091

Gra.1569.1.A1_at	Gorai.011G217200.1	bZIP			-0.465482728	-0.511789723
GhiAffx.19914.1.A1_s_at	Gorai.010G124200.1	bZIP			-0.617851274	-0.780542011
GhiAffx.22708.1.A1_at	Gorai.002G003000.1	bZIP				-0.835819152
Ghi.5630.1.S1_s_at	Gorai.010G008000.1	bZIP		-1.576502857	**-2.356608501**	-1.315021898
Ghi.5630.1.A1_s_at	Gorai.010G008000.1	bZIP	**-2.237794433**		-1.824400535	-1.316570252
GhiAffx.23589.1.S1_at	Gorai.013G035200.1	bZIP	**-2.33674547**	**-2.523240114**		-1.91543963
Ghi.8203.1.S1_x_at	Gorai.002G064900.1	bZIP			**-4.767501509**	**-3.55683861**
Gra.25.1.A1_s_at	Gorai.002G064900.1	bZIP			**-5.594007964**	**-4.005462518**
Ghi.5755.3.S1_s_at	Gorai.010G124200.1	bZIP			-0.722557357	
Ghi.9164.1.S1_s_at	Gorai.013G044500.1	bZIP		-0.800437267	-0.854941451	
Ghi.3805.1.A1_s_at	Gorai.007G277300.1	bZIP		**2.910353102**		
Ghi.10464.1.S1_at	Gorai.008G065300.1	bZIP		**2.023054705**		
Gra.1165.2.S1_s_at	Gorai.004G246700.1	bZIP		1.1125773		
GhiAffx.24063.1.S1_at	Gorai.008G068900.1	bZIP		-0.668542238		
GhiAffx.14224.2.S1_s_at	Gorai.013G000800.1	bZIP		-0.961270297		
Ghi.9296.1.S1_s_at	Gorai.009G212600.1	bZIP	1.103114101			
GhiAffx.6044.1.S1_at	Gorai.005G109600.1	bZIP	-1.482188671			
Ghi.1074.1.S1_s_at	Gorai.002G132600.1	C2H2				**4.779772145**
GhiAffx.3411.1.A1_at	Gorai.013G265200.1	C2H2	**3.202335541**	**2.958976267**	**3.265111427**	**4.383989923**
Ghi.807.1.S1_s_at	Gorai.013G050100.1	C2H2	1.749243894			**4.201321347**
Ghi.1999.1.A1_s_at	Gorai.005G232300.1	C2H2	**2.658641069**		**3.307238538**	**3.895113428**
Ghi.4983.1.A1_at	Gorai.002G132600.1	C2H2				**3.541440198**
Ghi.6901.1.A1_s_at	Gorai.008G020700.1	C2H2	**2.652687788**		**3.42449089**	**3.201646793**
Ghi.6704.1.S1_at	Gorai.008G082500.1	C2H2				**2.871834094**
Ghi.3286.1.S1_at	Gorai.010G025200.1	C2H2				**2.690003886**
Ghi.3160.1.A1_at	Gorai.003G136200.1	C2H2	**2.710716487**		**2.391270036**	**2.613876922**
Ghi.10485.1.S1_s_at	Gorai.009G218900.1	C2H2	**2.309120746**			**2.507916219**
GhiAffx.40824.1.A1_at	Gorai.010G202400.1	C2H2		**3.165023755**		**2.140636291**
GhiAffx.6350.1.S1_at	Gorai.005G186400.1	C2H2		1.71678229	1.286950839	1.783170229

GhiAffx.5728.1.A1_at	Gorai.007G298600.1	C2H2				0.953656175
GraAffx.1460.1.S1_at	Gorai.007G060600.1	C2H2	1.380801292		1.238854774	0.926256879
GhiAffx.33753.1.S1_at	Gorai.011G064700.1	C2H2				0.900718403
GhiAffx.28692.1.A1_s_at	Gorai.005G075000.1	C2H2				0.898823129
Ghi.2386.1.A1_s_at	Gorai.006G137400.1	C2H2	1.460135341		1.092641817	0.839924292
GhiAffx.46662.1.S1_at	Gorai.003G016200.1	C2H2		1.902970905	1.299270141	0.764574349
Ghi.8968.1.A1_s_at	Gorai.005G075000.1	C2H2				0.609211291
Ghi.9426.2.A1_s_at	Gorai.009G092800.1	C2H2			-0.71220303	-0.394277086
GhiAffx.22179.1.S1_x_at	Gorai.009G288800.1	C2H2				-0.400596931
Ghi.4720.1.A1_x_at	Gorai.002G214700.1	C2H2				-0.635285296
GhiAffx.45765.1.S1_at	Gorai.001G131000.1	C2H2	-1.158211786		-0.631804912	-0.701863178
GhiAffx.41382.1.A1_at	Gorai.003G099900.1	C2H2	-1.14178053			-0.880510705
Ghi.4440.1.A1_at	Gorai.002G123600.1	C2H2	**-3.569952953**		**-2.570366756**	-1.937053599
Gra.3014.1.S1_at	Gorai.003G016200.1	C2H2		**2.180123252**	1.21558176	
Ghi.2343.1.A1_at	Gorai.009G242400.1	C2H2	-0.773481478		-0.693009604	
Ghi.8315.1.S1_s_at	Gorai.009G195000.1	C2H2		-1.047025531	-0.940881838	
GhiAffx.2210.1.A1_at	Gorai.013G031600.1	C2H2			-1.007862942	
Gra.2370.1.S1_at	Gorai.008G168900.1	C2H2	-1.341422453		-1.09031107	
Ghi.3175.3.S1_at	Gorai.002G214700.1	C2H2		-0.678243291		
GhiAffx.22179.2.S1_x_at	Gorai.009G288800.1	C2H2		-0.858926901		
Ghi.9957.1.S1_at	Gorai.002G123600.1	C2H2		-0.876885135		
GraAffx.25536.1.A1_s_at	Gorai.007G371300.1	C2H2	1.465786757			
GraAffx.17159.1.A1_s_at	Gorai.002G214700.1	C2H2	1.099553916			
GraAffx.8466.1.S1_s_at	Gorai.013G031600.1	C2H2	-1.216083196			
Ghi.9149.1.A1_s_at	Gorai.013G017600.1	C3H			**3.448821755**	**3.424341585**
GhiAffx.3717.1.S1_s_at	Gorai.013G017600.1	C3H			**2.689642267**	**2.926002748**
Ghi.1708.1.S1_at	Gorai.007G197200.1	C3H	**3.330015432**	**2.352422894**	**2.449461374**	**2.750026698**
Ghi.1110.1.A1_s_at	Gorai.011G289400.1	C3H			1.299281748	1.470772422
Ghi.2685.1.S1_s_at	Gorai.010G196500.1	C3H				1.446385404

Gra.27.1.S1_s_at	Gorai.011G289400.1	C3H				1.420747523
GhiAffx.20832.1.S1_at	Gorai.003G011400.1	C3H				0.814157972
Ghi.5195.1.A1_s_at	Gorai.008G204400.1	C3H	-1.03765964			-0.500461419
Gra.354.1.S1_at	Gorai.001G009900.1	C3H				-0.806955824
GhiAffx.5509.1.S1_at	Gorai.009G029300.1	C3H	-1.18423134		-1.642377004	-0.958630262
Gra.280.1.A1_s_at	Gorai.004G288300.1	C3H			-0.834492133	-1.282378324
GraAffx.9235.1.S1_at	Gorai.013G229000.1	C3H		**-3.405529268**	-1.333312682	-1.689174904
Gra.355.1.S1_s_at	Gorai.001G009900.1	C3H		**-2.121630894**	-1.466669807	-1.861409586
Ghi.10383.1.S1_s_at	Gorai.013G229000.1	C3H	-1.738422806	**-2.427363124**	-1.846603496	-1.923443457
Gra.354.1.A1_at	Gorai.001G009900.1	C3H	**-2.558883045**	**-2.430767219**	-1.613416773	**-2.11776212**
Ghi.7498.3.S1_at	Gorai.005G129300.1	C3H			-0.578473956	
Ghi.8231.1.S1_s_at	Gorai.001G024400.1	C3H		-1.308169095	-0.790442258	
GhiAffx.26204.1.A1_at	Gorai.011G204700.1	CAMTA				0.827450527
Ghi.3380.2.A1_at	Gorai.008G089900.1	CAMTA		-0.921383703		
Gra.344.1.A1_x_at	Gorai.010G245200.1	CO-like				-0.373561039
Gra.2392.1.A1_s_at	Gorai.004G030300.1	CO-like			-0.682546267	-0.565459659
Ghi.10472.2.S1_s_at	Gorai.009G065600.1	CO-like	-1.051431804			-0.578258278
GhiAffx.22893.1.A1_s_at	Gorai.004G102000.1	CO-like	-1.997657625	-0.688256747	-1.50264929	-0.984322467
GraAffx.10337.1.A1_at	Gorai.004G102000.1	CO-like		-0.896560443	-1.431134928	-0.993524051
Ghi.10472.2.A1_s_at	Gorai.009G065600.1	CO-like			-1.153043724	-1.076740051
GraAffx.5920.1.A1_at	Gorai.006G061600.1	CO-like	**-2.756284627**	**-2.307236179**	-1.986026141	-1.195323799
GraAffx.2336.1.S1_at	Gorai.013G246000.1	CO-like			**-2.649322947**	**-3.132676708**
GhiAffx.15731.1.S1_s_at	Gorai.002G022300.1	CO-like		**3.045248537**		
Gra.2392.1.S1_s_at	Gorai.004G030300.1	CO-like	-0.870747407			
Gra.344.2.S1_x_at	Gorai.010G245200.1	CO-like	**-2.129640075**			
Ghi.2046.1.S1_s_at	Gorai.006G229300.1	DBB	-1.272211376		-0.420359497	-0.296504277
Gra.656.2.S1_at	Gorai.006G229300.1	DBB	-1.264522662		-0.728993944	
GhiAffx.44888.1.S1_at	Gorai.009G139800.1	Dof		1.902602028	1.088403853	1.58295715
GhiAffx.7142.1.S1_at	Gorai.004G012700.1	Dof				1.498913958

GhiAffx.16775.1.A1_at	Gorai.004G252700.1	Dof				0.65512997
GhiAffx.5701.1.S1_at	Gorai.011G067500.1	Dof		1.536645339		0.483644515
GraAffx.16567.1.A1_x_at	Gorai.001G109300.1	Dof			-0.721813369	-0.647504991
Ghi.5254.1.A1_at	Gorai.010G159300.1	Dof				-0.826899753
GhiAffx.52921.1.A1_at	Gorai.001G067000.1	Dof	-1.813740464		-1.594054832	-0.863849929
GhiAffx.46630.1.S1_at	Gorai.007G040400.1	Dof			-1.398417608	-1.151726903
Ghi.4728.1.S1_at	Gorai.007G079300.1	Dof	-0.586533403		-1.102206533	-1.317668246
Ghi.4728.1.A1_at	Gorai.007G079300.1	Dof				-1.480908695
GhiAffx.22038.1.A1_at	Gorai.009G168300.1	Dof	-1.561465691		-0.873217128	
GhiAffx.25540.1.A1_s_at	Gorai.008G193700.1	Dof	**-2.539187422**			
GhiAffx.16184.1.S1_at	Gorai.004G227800.1	EIL	1.32921958	1.280185684	1.907588247	1.450078002
Ghi.7115.1.S1_s_at	Gorai.004G227800.1	EIL				0.704801709
Gra.260.2.S1_s_at	Gorai.004G227800.1	EIL			0.822130112	0.555479154
GhiAffx.43606.1.S1_s_at	Gorai.013G266300.1	EIL		0.527301825		
Ghi.4920.1.A1_at	Gorai.001G016800.1	ERF	**3.421099602**	**4.872124327**		**6.730743365**
GhiAffx.15854.1.S1_at	Gorai.008G025100.1	ERF	**5.383905183**		**4.437813529**	**6.026687968**
Ghi.9720.1.S1_at	Gorai.013G043700.1	ERF				**5.665476613**
Ghi.3673.1.S1_at	Gorai.010G091100.1	ERF				**5.615286355**
GhiAffx.59715.1.S1_at	Gorai.001G019100.1	ERF	**6.621653758**	**3.827098458**	**3.549604422**	**4.786970057**
GhiAffx.25508.1.S1_at	Gorai.008G075600.1	ERF	**5.868899235**	**5.374549915**		**4.238026515**
Ghi.6808.1.S1_s_at	Gorai.002G067600.1	ERF	**2.235842317**			**4.132598467**
GhiAffx.44148.1.S1_at	Gorai.003G048800.1	ERF	**5.856747471**	**4.234388111**	**3.447621007**	**3.843098639**
Ghi.8116.1.S1_at	Gorai.008G280600.1	ERF				**3.579808662**
GhiAffx.60792.1.S1_at	Gorai.006G197200.1	ERF	**5.262016016**	**3.020922797**	**3.226434192**	**3.428159194**
Ghi.9959.1.S1_s_at	Gorai.011G029800.1	ERF	1.790192418			**2.89981174**
Ghi.162.1.S1_s_at	Gorai.011G029800.1	ERF				**2.838801448**
Ghi.5157.1.S1_s_at	Gorai.005G200200.1	ERF				**2.712207099**
Gra.1495.1.S1_s_at	Gorai.011G029800.1	ERF				**2.646897739**
Ghi.9726.2.S1_s_at	Gorai.010G155900.1	ERF				**2.597017401**

Ghi.9726.1.A1_s_at	Gorai.010G155900.1	ERF				**2.589764273**
Ghi.10747.1.S1_at	Gorai.006G214100.1	ERF			**3.712804351**	**2.4037786**
Ghi.8126.1.S1_x_at	Gorai.009G375700.1	ERF				**2.327136554**
Ghi.9880.2.S1_at	Gorai.003G176200.1	ERF				**2.23497058**
Ghi.8749.1.S1_at	Gorai.008G232700.1	ERF				**2.200751836**
GhiAffx.1517.1.S1_at	Gorai.004G051600.1	ERF			1.502490828	**2.095717455**
Ghi.9124.3.A1_at	Gorai.009G147300.1	ERF		1.73836128	**2.402775225**	**2.093677701**
Ghi.10481.2.S1_s_at	Gorai.004G142800.1	ERF				**2.086232304**
GhiAffx.6784.1.A1_at	Gorai.007G026200.1	ERF	**2.129098395**	1.735621346		**2.039463788**
Ghi.10481.3.S1_s_at	Gorai.004G142800.1	ERF				1.980206694
Ghi.9880.1.A1_s_at	Gorai.003G176200.1	ERF				1.857522491
Ghi.4716.1.A1_s_at	Gorai.013G076100.1	ERF				1.849551296
Ghi.9880.2.A1_x_at	Gorai.003G176200.1	ERF				1.749269345
GhiAffx.59933.1.S1_at	Gorai.009G266200.1	ERF	**4.117219761**			1.716541038
Ghi.9749.1.S1_s_at	Gorai.011G113300.1	ERF				1.660142492
Ghi.10481.3.A1_at	Gorai.004G142800.1	ERF		0.653767666		1.586497157
GhiAffx.39399.1.S1_at	Gorai.010G156600.1	ERF		**2.481204692**	**2.028821293**	1.554267525
Ghi.9175.1.S1_at	Gorai.003G112200.1	ERF				1.505936618
Ghi.9501.1.S1_at	Gorai.011G029700.1	ERF	1.89789779		1.522105617	1.283456252
Ghi.3215.1.S1_s_at	Gorai.009G238800.1	ERF		1.262959951		1.238279675
GraAffx.3551.1.A1_at	Gorai.008G232700.1	ERF				1.236166818
Ghi.9124.1.S1_x_at	Gorai.009G147300.1	ERF				1.167409153
Ghi.3846.1.S1_at	Gorai.003G038200.1	ERF		**2.30879128**		1.105342759
Ghi.2696.2.S1_s_at	Gorai.010G043200.1	ERF				1.072942998
GhiAffx.60224.1.S1_s_at	Gorai.007G240700.1	ERF		0.963413474		1.04329763
Gra.894.1.A1_at	Gorai.003G112200.1	ERF				1.020819747
Ghi.9124.2.S1_s_at	Gorai.009G147300.1	ERF				0.954118063
GraAffx.26388.2.A1_x_at	Gorai.010G043200.1	ERF		1.209995347		0.946409871
Gra.796.1.A1_at	Gorai.011G113300.1	ERF				0.904302399

Gra.2756.1.S1_x_at	Gorai.009G147300.1	ERF				0.812556554
GhiAffx.13016.1.S1_x_at	Gorai.010G043200.1	ERF		0.72372979		0.657536197
Ghi.3913.1.S1_at	Gorai.005G093500.1	ERF				0.57029068
Ghi.3636.1.A1_s_at	Gorai.008G183300.1	ERF				0.567332207
Ghi.5532.1.S1_s_at	Gorai.010G236100.1	ERF				0.42648267
Ghi.3095.2.S1_s_at	Gorai.008G077900.1	ERF	0.659149524			-0.869362549
Ghi.2915.1.A1_at	Gorai.009G443400.1	ERF	-0.774627395	-0.738815869	-0.872912022	-1.009744258
GbaAffx.196.1.A1_at	Gorai.006G260300.1	ERF	**2.029869682**		**2.88535003**	-1.451946368
Ghi.8128.1.S1_x_at	Gorai.006G260300.1	ERF	1.053201315		1.722250542	-1.468262156
Ghi.7874.1.S1_s_at	Gorai.006G260300.1	ERF	0.976616197		1.548344371	-1.600531921
GbaAffx.196.1.A1_s_at	Gorai.006G260300.1	ERF	0.967144726		1.594892686	-1.630028523
GhiAffx.4057.1.A1_at	Gorai.010G114800.1	ERF			**-3.892318326**	**-4.884250044**
GhiAffx.24637.1.S1_at	Gorai.011G173000.1	ERF			**4.558347721**	
Ghi.6885.1.A1_s_at	Gorai.009G087700.1	ERF	**6.773599943**	**3.79524524**	**3.717152657**	
Ghi.8161.1.S1_s_at	Gorai.008G232500.1	ERF			**2.89855964**	
Ghi.2255.1.S1_at	Gorai.011G193600.1	ERF	1.851013211		**2.044118482**	
Gra.302.1.S1_s_at	Gorai.011G193600.1	ERF	1.417644072	1.414024854	1.863326545	
Ghi.4480.1.S1_s_at	Gorai.011G056300.1	ERF			0.662591456	
Gra.2596.1.S1_s_at	Gorai.011G056300.1	ERF			0.527029023	
Ghi.3846.1.A1_s_at	Gorai.003G038200.1	ERF		1.893478352		
Ghi.2696.1.A1_s_at	Gorai.010G043200.1	ERF		1.028940732		
Ghi.9501.1.A1_s_at	Gorai.011G029700.1	ERF	1.293518872			
GhiAffx.7865.1.S1_at	Gorai.007G046100.1	ERF	-1.695037801			
GhiAffx.28739.1.S1_s_at	Gorai.007G046000.1	ERF	**-2.278635979**			
Ghi.4571.1.A1_at	Gorai.003G140100.1	FAR1		**4.287289953**	**2.897263229**	**2.776288123**
GhiAffx.46871.1.S1_at	Gorai.003G140100.1	FAR1	-1.162661906		-0.630656749	-0.625847946
GhiAffx.34280.1.A1_at	Gorai.005G126700.1	FAR1		-0.692493287		
GhiAffx.2392.1.S1_at	Gorai.013G065300.1	FAR1	-0.71525467			
GhiAffx.26670.1.S1_s_at	Gorai.007G173400.1	FAR1	-0.730403967			

GhiAffx.8670.1.A1_at	Gorai.009G127000.1	FAR1	-1.183031135			
Gra.2977.1.S1_at	Gorai.003G096100.1	FAR1	-1.405522904			
Ghi.501.2.S1_at	Gorai.008G185500.1	G2-like		1.451952102	**2.169683935**	**2.105351044**
Ghi.501.2.A1_at	Gorai.008G185500.1	G2-like			1.774027952	1.864073802
GhiAffx.39948.1.S1_at	Gorai.008G073300.1	G2-like		1.344776971	1.787996955	1.809060581
GhiAffx.59955.1.S1_at	Gorai.003G021300.1	G2-like		1.299363758	1.808696639	1.618209551
GhiAffx.3456.1.A1_a_at	Gorai.009G120900.1	G2-like	-1.490338661	**-2.073285721**	-1.843948455	-0.98931277
GraAffx.1960.1.S1_at	Gorai.007G287500.1	G2-like				-1.118761077
GhiAffx.40609.1.S1_at	Gorai.005G033900.1	G2-like			-1.57372351	
Gra.1734.1.A1_s_at	Gorai.006G222400.1	G2-like		-0.842290598		
Ghi.5072.1.A1_s_at	Gorai.012G150500.1	G2-like	**-2.489157201**	-1.028397965		
GhiAffx.6545.1.S1_at	Gorai.008G155000.1	G2-like		-1.640582		
Gra.42.1.S1_s_at	Gorai.009G313700.1	GATA				**2.776376909**
Ghi.10341.1.A1_s_at	Gorai.007G049700.1	GATA				1.973806059
GraAffx.26496.1.S1_x_at	Gorai.011G238800.1	GATA	**2.318195289**			1.878215094
GhiAffx.34581.1.A1_x_at	Gorai.011G238800.1	GATA	**2.051401582**			1.796387302
Ghi.10341.2.A1_x_at	Gorai.007G049700.1	GATA			0.636559504	1.118602361
Ghi.9110.1.S1_at	Gorai.013G032900.1	GATA				0.656523391
Ghi.10341.1.S1_s_at	Gorai.007G049700.1	GATA				0.635487786
GhiAffx.48282.1.S1_a_at	Gorai.005G097100.1	GATA				-0.716536093
GhiAffx.21747.1.S1_at	Gorai.013G127900.1	GATA	-1.31489218	**-2.314279828**	-1.335223641	-0.89421805
GhiAffx.61075.1.S1_s_at	Gorai.004G230100.1	GATA	-1.462569126		**-2.162399131**	-1.767114297
GraAffx.29709.1.A1_s_at	Gorai.009G031400.1	GATA	-1.724734458	-0.853010681	-1.111090399	
GhiAffx.21686.1.S1_at	Gorai.010G101100.1	GATA	**-2.640238962**		-1.463675117	
GhiAffx.1175.1.S1_at	Gorai.008G231500.1	GATA	-1.039241452			
Ghi.986.1.S1_at	Gorai.013G121800.1	GeBP		0.728539851	1.089756364	1.484329459
GhiAffx.60941.1.S1_at	Gorai.001G117300.1	GeBP			-0.527793776	-0.720635663
Ghi.3057.1.S1_at	Gorai.008G058900.1	GeBP		-0.6199978		
Ghi.10329.1.S1_at	Gorai.009G427800.1	GRAS	**3.779589673**	**2.964266216**	**3.363434533**	**3.582696804**

Ghi.3132.1.S1_at	Gorai.010G249400.1	GRAS				**2.660331751**
Ghi.9123.1.S1_at	Gorai.003G130400.1	GRAS				1.517840808
Ghi.3126.1.S1_s_at	Gorai.002G096300.1	GRAS	**2.274626124**	1.971897161		1.479111075
GhiAffx.44794.1.S1_at	Gorai.009G098100.1	GRAS	**2.000341586**		**3.232093214**	1.423669682
Ghi.9302.1.A1_at	Gorai.005G083700.1	GRAS	1.315146201		1.299853169	1.24434727
GhiAffx.63632.1.S1_at	Gorai.006G198300.1	GRAS		1.127866121		1.171120826
Ghi.4551.1.A1_at	Gorai.005G014200.1	GRAS	1.218054422			1.136416022
Ghi.4703.1.A1_s_at	Gorai.002G096300.1	GRAS	1.663958505	1.382270955		0.963642461
Ghi.4703.1.S1_at	Gorai.002G096300.1	GRAS	1.49558182	1.195059656		0.942174055
Ghi.4006.1.S1_s_at	Gorai.001G089400.1	GRAS	-1.61542623		-1.054527841	-0.807517263
Ghi.9219.2.A1_s_at	Gorai.007G167000.1	GRAS	**-2.047664108**		-1.540666759	-0.859790645
GhiAffx.11496.1.A1_at	Gorai.003G030400.1	GRAS				-0.877927808
GhiAffx.16118.1.S1_s_at	Gorai.001G089400.1	GRAS	-1.829520011		-1.12612192	-0.888760338
GhiAffx.34665.1.S1_at	Gorai.009G021700.1	GRAS			**-4.407268683**	**-2.65788116**
Ghi.9360.1.S1_at	Gorai.002G080600.1	GRAS	**3.484284291**	**2.374356215**		
Gra.1358.1.A1_at	Gorai.013G164700.1	GRAS	**3.535527424**			
Ghi.9360.1.A1_at	Gorai.002G080600.1	GRAS	1.449404492			
GraAffx.21485.1.A1_s_at	Gorai.002G096300.1	GRAS	1.352401852			
GhiAffx.44405.1.A1_at	Gorai.003G130600.1	GRAS	-1.406040328			
GhiAffx.3767.1.S1_at	Gorai.011G266200.1	GRAS	-1.69862948			
GhiAffx.23338.1.S1_at	Gorai.008G068100.1	GRAS	**-2.557899656**			
GhiAffx.23038.1.A1_at	Gorai.007G092400.1	GRF	-1.02071974		-0.947011741	
GhiAffx.18483.1.S1_at	Gorai.010G027700.1	GRF	**-3.254154468**		**-3.531114324**	
Ghi.4893.2.A1_x_at	Gorai.010G256900.1	HB-other				0.643549624
Ghi.8823.1.S1_at	Gorai.009G451700.1	HB-other				0.536363594
Ghi.4147.1.S1_at	Gorai.004G014700.1	HB-other		-0.905831404	-1.179835833	-0.660869247
GraAffx.33997.1.A1_s_at	Gorai.004G274200.1	HB-other	-0.863329238			
GhiAffx.43409.1.S1_at	Gorai.012G148900.1	HB-PHD	1.633094318		1.235561236	1.521447653
GhiAffx.6490.1.S1_at	Gorai.001G256300.1	HD-ZIP				1.016865777

GhiAffx.17252.1.A1_at	Gorai.009G049100.1	HD-ZIP			-1.491929419	-0.818068954
Ghi.9687.1.S1_s_at	Gorai.011G111500.1	HD-ZIP	**-2.886660802**	-1.801969246	-1.566643353	-0.826222863
Ghi.9416.1.S1_s_at	Gorai.008G233200.1	HD-ZIP	-1.815708339		**-2.273833052**	-1.053273223
Gra.2833.1.S1_at	Gorai.003G041500.1	HD-ZIP	**-2.856042192**	-1.165882266	-1.876802066	-1.141333303
Ghi.9416.2.A1_s_at	Gorai.008G233200.1	HD-ZIP	-1.285251796		**-2.345286391**	-1.176857232
GraAffx.19826.1.A1_x_at	Gorai.003G094500.1	HD-ZIP	**-2.33660799**	-1.229677885		-1.346330254
GhiAffx.21613.1.S1_s_at	Gorai.005G139600.1	HD-ZIP			-1.832262761	-1.416264159
GhiAffx.25293.1.S1_s_at	Gorai.007G029200.1	HD-ZIP	**-3.041447905**	-1.244738705	**-2.830084769**	-1.527654455
Ghi.9572.1.S1_s_at	Gorai.009G323600.1	HD-ZIP			**-2.460414573**	-1.588885154
GraAffx.34036.1.S1_s_at	Gorai.011G111500.1	HD-ZIP	**-2.188996735**	-1.836394909	-1.880131862	-1.599541166
GhiAffx.43198.1.S1_at	Gorai.005G189800.1	HD-ZIP	-1.428762575	-1.926965488	**-2.009655445**	-1.641125353
GhiAffx.47045.1.S1_s_at	Gorai.011G004200.1	HD-ZIP			-1.492412119	
GhiAffx.27818.1.S1_at	Gorai.005G150100.1	HD-ZIP		-1.291644419		
GraAffx.21312.2.S1_s_at	Gorai.009G049100.1	HD-ZIP	-0.760695099			
GraAffx.10252.1.A1_at	Gorai.008G190300.1	HD-ZIP	-1.597637186			
GhiAffx.12134.1.S1_at	Gorai.008G172800.1	HD-ZIP	-1.687838371			
GraAffx.28942.1.S1_s_at	Gorai.003G023500.1	HSF			**2.931425088**	**2.356586259**
GhiAffx.58009.1.S1_at	Gorai.001G012700.1	HSF				1.392451022
Ghi.6713.1.S1_s_at	Gorai.006G158000.1	HSF	1.794814881			0.80643048
GraAffx.12789.1.A1_s_at	Gorai.004G208800.1	HSF		-0.84705586		0.485647299
Ghi.3792.1.A1_at	Gorai.003G053300.1	HSF				-0.595856065
Ghi.10292.1.S1_s_at	Gorai.013G220400.1	HSF		**-2.416547718**		-0.696593796
GhiAffx.63874.1.S1_at	Gorai.010G070900.1	HSF	-1.434528085			
GhiAffx.30354.1.S1_at	Gorai.008G056000.1	LBD	**2.521239099**	**3.350622179**	**2.488363008**	**3.622537906**
GhiAffx.44180.1.S1_at	Gorai.010G170700.1	LBD				1.224177151
GhiAffx.12107.1.S1_at	Gorai.010G203800.1	LBD		1.203552764		
Gra.2751.1.S1_s_at	Gorai.008G227400.1	LSD	**-3.268676332**	-1.402476457	-1.961940614	-0.724995963
Gra.2751.2.A1_a_at	Gorai.008G227400.1	LSD	**-3.544839404**		**-2.891214027**	-1.483934177
GhiAffx.32507.1.S1_at	Gorai.004G186300.1	LSD	**-2.719516701**			

GraAffx.32054.1.S1_at	Gorai.003G011500.1	MIKC				-0.672270247
GhiAffx.10806.1.S1_at	Gorai.005G225500.1	MIKC	-0.708856691			
Ghi.1693.1.S1_x_at	Gorai.001G075800.1	MIKC	-1.15236279			
GhiAffx.60040.1.S1_s_at	Gorai.013G210600.1	M-type		-1.513009176	-1.849309262	-1.07813557
GhiAffx.21099.1.S1_at	Gorai.004G145800.1	MYB	**4.900226929**	**3.891287412**	**3.852283485**	**4.686276089**
Ghi.6693.1.A1_at	Gorai.006G060300.1	MYB	**4.677211649**	**5.03952994**	**5.785938809**	**4.2833477**
Ghi.8089.1.S1_at	Gorai.007G121000.1	MYB	**4.162483702**	**3.692186908**	**2.863442141**	**3.14193009**
Ghi.10620.1.S1_at	Gorai.009G208900.1	MYB	1.856761138	1.635275204	**2.8527486**	**2.951873062**
Ghi.3797.1.S1_at	Gorai.008G060100.1	MYB	**3.148734338**		**2.773310805**	**2.659043125**
Ghi.3797.1.A1_at	Gorai.008G060100.1	MYB	**3.496133385**		**3.064173543**	**2.611432877**
GhiAffx.48583.1.S1_at	Gorai.005G068900.1	MYB	**2.307901731**	**2.89503526**	1.921114131	**2.368105946**
Ghi.6693.1.S1_at	Gorai.006G060300.1	MYB	**2.80759202**	**3.553435192**	**2.770309172**	**2.112646868**
Ghi.9150.1.S1_at	Gorai.009G174500.1	MYB	**2.281477945**	**2.705668406**		1.710744699
GhiAffx.13694.1.S1_x_at	Gorai.010G212600.1	MYB			1.460716464	1.453010207
GhiAffx.8694.1.S1_a_at	Gorai.004G029000.1	MYB	**3.698072704**	**3.872774152**		1.420446624
Ghi.9954.2.A1_s_at	Gorai.007G218100.1	MYB				1.412783443
Ghi.9954.1.S1_s_at	Gorai.007G218100.1	MYB				1.094462216
Ghi.9740.2.A1_at	Gorai.007G346400.1	MYB				0.993636571
Ghi.9209.1.S1_at	Gorai.007G055600.1	MYB				0.925792042
GraAffx.34168.1.S1_s_at	Gorai.007G218100.1	MYB				0.750936426
Gra.1024.1.S1_at	Gorai.012G135600.1	MYB				-0.624115273
Ghi.8076.1.S1_at	Gorai.007G350500.1	MYB				-0.642384376
Gra.1546.1.A1_s_at	Gorai.013G067600.1	MYB			-0.943144323	-0.907094329
Ghi.9407.1.S1_s_at	Gorai.012G135600.1	MYB	-1.51650203		-0.970609072	-0.95255321
Ghi.8082.1.S1_s_at	Gorai.002G196700.1	MYB			-1.218138087	-1.267615536
Ghi.4770.1.S1_at	Gorai.002G261600.1	MYB	**-2.070871935**			-1.28709483
Ghi.5933.1.S1_s_at	Gorai.013G067600.1	MYB	-0.875393825		-1.502167224	-1.308199094
Ghi.591.1.S1_at	Gorai.012G112000.1	MYB	-1.03670922		-1.509323584	-1.385230859
Gra.641.1.S1_at	Gorai.006G063000.1	MYB	-0.862593735	**-2.377315675**	**-2.123753285**	-1.494458015

GhiAffx.26171.1.A1_at	Gorai.013G196800.1	MYB	-1.772324127		-1.957614069	-1.613164586
GhiAffx.21415.1.S1_at	Gorai.004G201900.1	MYB		-1.557720615	**-2.071146916**	-1.790726898
Ghi.8080.1.S1_at	Gorai.001G020600.1	MYB		-1.578163889		**-2.64316573**
Ghi.2459.1.S1_at	Gorai.012G052500.1	MYB	**-2.915784447**		**-3.089853916**	**-2.666050925**
Ghi.8087.1.S1_s_at	Gorai.009G146500.1	MYB	-1.337119095			**-2.927923424**
GarAffx.37322.1.S1_s_at	Gorai.004G196800.1	MYB			-1.672417678	**-2.972382416**
Ghi.9272.1.S1_s_at	Gorai.009G263200.1	MYB			1.544982924	
GhiAffx.46539.1.S1_at	Gorai.008G192900.1	MYB	-1.633191116		-0.921023062	
Gra.1546.1.S1_s_at	Gorai.013G067600.1	MYB			-1.188316346	
GraAffx.5629.1.A1_at	Gorai.013G196800.1	MYB			-1.37781462	
GhiAffx.18330.1.S1_s_at	Gorai.008G035700.1	MYB			-1.501062122	
Ghi.10620.3.A1_at	Gorai.009G208900.1	MYB		1.088375552		
GraAffx.24514.1.A1_at	Gorai.001G020500.1	MYB		-1.206296994		
Ghi.3201.1.S1_s_at	Gorai.005G234900.1	MYB	**2.797839852**			
GhiAffx.15325.1.S1_at	Gorai.006G180900.1	MYB	1.851771728			
GraAffx.8123.1.A1_at	Gorai.008G192900.1	MYB	-1.170762751			
GhiAffx.5921.1.S1_at	Gorai.011G279700.1	MYB_related	-1.911391988	-1.79876236		-0.548630004
Ghi.1711.1.S1_s_at	Gorai.007G371900.1	MYB_related	-1.887087695		-0.752099663	-0.893351803
Ghi.2174.1.S1_s_at	Gorai.006G178000.1	MYB_related			-1.221876734	-0.907437309
GhiAffx.13229.1.A1_at	Gorai.002G083400.1	MYB_related				**-2.018053088**
GhiAffx.22445.1.S1_at	Gorai.013G036700.1	MYB_related			-1.284804587	**-2.695220191**
GhiAffx.24680.1.S1_at	Gorai.002G127300.1	MYB_related				**-2.719854433**
Ghi.1719.1.S1_at	Gorai.007G224200.1	MYB_related	-1.300209835	-0.823057482	-0.688247309	
Gra.861.1.A1_at	Gorai.010G081800.1	MYB_related			-0.856441016	
Ghi.1098.2.S1_s_at	Gorai.007G034000.1	MYB_related		-0.899350355		
Ghi.4471.1.A1_at	Gorai.009G201700.1	MYB_related	**2.922798593**			
GhiAffx.23270.1.S1_at	Gorai.006G142300.1	MYB_related	-0.827846034			
GhiAffx.17808.1.S1_at	Gorai.010G081800.1	MYB_related	-1.59009886			
Ghi.3801.1.A1_at	Gorai.006G113000.1	NAC	**5.267704307**	**4.261757938**	**5.921704761**	**6.86432376**

Ghi.3801.1.S1_s_at	Gorai.006G113000.1	NAC	**4.798983006**	**3.732892281**	**4.983242858**	**5.598858608**
GhiAffx.58305.1.S1_s_at	Gorai.012G037600.1	NAC	**2.518891552**		**4.009254692**	**3.610899068**
Ghi.7907.1.S1_s_at	Gorai.002G073700.1	NAC		**2.281245108**	**3.63267754**	**2.780716008**
Gra.1120.1.S1_s_at	Gorai.002G073700.1	NAC		**2.618508416**	**3.402733227**	**2.666147743**
GhiAffx.33994.1.A1_s_at	Gorai.007G267900.1	NAC			**3.612920471**	**2.412723597**
Ghi.7907.2.A1_at	Gorai.002G073700.1	NAC		1.655170908	**3.238146977**	**2.219936899**
Ghi.3574.1.S1_s_at	Gorai.005G195300.1	NAC	**2.564119149**	**2.187012091**	**2.666017368**	**2.118253342**
GhiAffx.20354.1.S1_at	Gorai.005G195400.1	NAC	**2.386723553**	**2.407203358**	**2.743056944**	1.886675321
GhiAffx.63871.1.S1_at	Gorai.004G221300.1	NAC				1.873586514
GhiAffx.62090.1.A1_s_at	Gorai.006G203800.1	NAC	**2.095871859**		1.730794968	1.851538628
Gra.450.1.S1_at	Gorai.007G017500.1	NAC			1.188851006	1.850931694
GhiAffx.34015.1.S1_at	Gorai.004G186700.1	NAC	-1.549868545			1.707811529
GraAffx.7931.1.A1_at	Gorai.012G083700.1	NAC	**2.108111728**		1.504688264	1.698315266
Ghi.10004.1.S1_at	Gorai.007G017500.1	NAC				1.593554977
GraAffx.17500.1.A1_s_at	Gorai.007G267900.1	NAC				1.38511145
Ghi.3264.2.A1_s_at	Gorai.010G051900.1	NAC	**2.364212688**	1.263161052	1.400656036	1.268210313
GhiAffx.6415.1.S1_s_at	Gorai.004G125900.1	NAC			0.875938488	1.126879399
Ghi.3264.1.S1_s_at	Gorai.010G051900.1	NAC	**2.029959084**	1.026247392		1.087206517
GhiAffx.49721.1.A1_at	Gorai.001G231400.1	NAC				0.503315529
Ghi.6896.1.A1_s_at	Gorai.003G003900.1	NAC		-1.695616368	**2.617248545**	
Ghi.7468.1.S1_at	Gorai.007G267700.1	NAC			**2.242985124**	
Ghi.6896.1.S1_at	Gorai.003G003900.1	NAC	1.022113635	-1.026346963	1.961999311	
Gra.1281.1.A1_s_at	Gorai.013G191800.1	NAC			1.129262355	
Gra.2413.1.A1_s_at	Gorai.010G051900.1	NAC	**2.456643251**		1.056864489	
GraAffx.14152.1.S1_s_at	Gorai.005G088800.1	NAC	**2.916439389**	**2.137662844**		
Ghi.868.1.S1_x_at	Gorai.001G150000.1	NAC		-1.180629472		
Ghi.9283.2.A1_s_at	Gorai.007G114500.1	NAC		**-2.114332698**		
Ghi.9283.3.A1_at	Gorai.007G114500.1	NAC	-1.016944775			
Ghi.616.1.S1_at	Gorai.007G038100.1	NAC	-1.264477752			

Ghi.9328.1.S1_s_at	Gorai.012G125500.1	NAC	-1.506599219			
GraAffx.29995.1.S1_s_at	Gorai.012G125500.1	NAC	-1.740533654			
Ghi.3035.1.A1_at	Gorai.006G058600.1	NF-X1				1.118953462
GhiAffx.24236.1.S1_at	Gorai.011G004400.1	NF-YA	-0.729420939			
GhiAffx.22229.4.S1_at	Gorai.001G222300.1	NF-YB			-0.617240331	-0.68467446
Gra.332.1.A1_at	Gorai.013G153100.1	NF-YB	-1.190396204		-1.608801653	
Ghi.5325.1.A1_at	Gorai.007G336800.1	NF-YC				0.71744695
Gra.286.1.S1_s_at	Gorai.001G058500.1	NF-YC			-0.658828615	
Ghi.9838.1.S1_s_at	Gorai.001G058500.1	NF-YC			-0.845005205	
GhiAffx.44010.1.A1_s_at	Gorai.002G110200.1	NF-YC		-1.057498487		
Ghi.3384.3.S1_at	Gorai.008G041100.1	Nin-like	**-2.159775142**		**-2.172026044**	**-2.057327901**
Ghi.2294.2.A1_at	Gorai.002G206700.1	Nin-like			1.38401737	
Ghi.3331.1.A1_s_at	Gorai.002G206700.1	Nin-like			1.119278291	
Ghi.3331.1.S1_s_at	Gorai.002G206700.1	Nin-like			0.806785789	
GhiAffx.9036.1.A1_at	Gorai.005G013500.1	Nin-like	-0.953325329			
GhiAffx.30941.1.S1_s_at	Gorai.008G185900.1	RAV				1.064394239
Ghi.8341.1.S1_x_at	Gorai.008G185900.1	RAV				0.804899101
Gra.876.1.A1_at	Gorai.005G138100.1	RAV				-0.64483377
GhiAffx.60824.1.S1_x_at	Gorai.005G138100.1	RAV				-0.775311559
Ghi.5715.1.S1_s_at	Gorai.006G168100.1	S1Fa-like				-0.543534631
GhiAffx.23747.1.S1_at	Gorai.003G003200.1	SBP	-1.258964596			-0.862098631
GhiAffx.130.1.A1_at	Gorai.012G176500.1	SBP		-1.377323261		-0.918700866
Ghi.8863.1.A1_at	Gorai.006G066700.1	SBP	-0.922852312		-0.959294526	-0.961941713
Ghi.8863.1.S1_s_at	Gorai.006G066700.1	SBP	-1.175960625		-1.055838245	-1.061929333
GhiAffx.17946.1.A1_at	Gorai.011G029400.1	SBP				-1.432030474
Ghi.8127.1.S1_s_at	Gorai.013G169800.1	SBP			**-3.142455801**	**-2.172036494**
Ghi.1896.1.S1_x_at	Gorai.012G176500.1	SBP	**2.465347342**		0.792290873	
GraAffx.26308.1.A1_s_at	Gorai.012G176500.1	SBP	**3.149195667**			
GhiAffx.22725.1.S1_at	Gorai.001G134100.1	SRS				0.616068101

GhiAffx.6612.1.A1_at	Gorai.007G198100.1	SRS			-0.705813109	-0.574104155
Gra.65.1.A1_s_at	Gorai.006G149100.1	TALE				0.508074244
Gra.714.1.S1_at	Gorai.009G396900.1	TALE				0.435417151
GraAffx.26728.1.S1_at	Gorai.009G012300.1	TALE			-0.449075511	-0.695546268
GhiAffx.3403.1.S1_at	Gorai.003G143600.1	TALE	**-2.30212535**		-1.869970757	-1.466281201
GraAffx.29310.1.S1_s_at	Gorai.004G206600.1	TALE			-0.559798979	
GraAffx.33424.1.A1_at	Gorai.003G156000.1	TALE	-1.780610067	-1.080265315		
GraAffx.9648.1.S1_s_at	Gorai.010G117100.1	TALE		-1.253087697		
GhiAffx.7276.1.S1_s_at	Gorai.007G207200.1	TALE	-1.42366005			
Ghi.1362.1.S1_at	Gorai.001G125400.1	TALE	-1.673264522			
Ghi.3203.1.A1_at	Gorai.002G064500.1	TCP		-0.943284879		-0.505873848
Gra.513.1.S1_s_at	Gorai.009G289000.1	TCP			-1.090572518	-0.537456902
GhiAffx.21807.1.S1_s_at	Gorai.006G197000.1	TCP				-0.657431989
Gra.813.1.S1_at	Gorai.002G064500.1	TCP				-1.00349724
Gra.513.1.A1_s_at	Gorai.009G289000.1	TCP	**-2.028149397**		-1.537798805	-1.252454488
Ghi.5000.1.S1_s_at	Gorai.007G036800.1	TCP	-1.716638037		-1.793266161	-1.767785326
GhiAffx.2605.1.S1_at	Gorai.008G181600.1	TCP			-0.579339803	
GhiAffx.56819.1.S1_at	Gorai.008G157300.1	TCP		-0.912735387		
GhiAffx.1589.25.S1_s_at	Gorai.012G084600.1	TCP		-1.186516754		
Ghi.8641.1.A1_at	Gorai.006G009800.1	TCP	-0.884808513			
GraAffx.7835.1.A1_at	Gorai.011G216400.1	Trihelix			-0.783900888	
GhiAffx.42556.1.S1_at	Gorai.007G283800.1	Trihelix	**-2.342719581**		-1.810906576	
Ghi.407.1.S1_s_at	Gorai.011G076900.1	Trihelix	-0.877091439			
GhiAffx.5960.2.S1_a_at	Gorai.004G167100.1	Trihelix	-0.922317394			
GhiAffx.43699.1.A1_at	Gorai.001G059600.1	Trihelix	-1.415594421			
Ghi.470.1.S1_at	Gorai.009G253700.1	Trihelix	-1.516274213			
GhiAffx.41414.1.A1_at	Gorai.009G253900.1	Trihelix	**-2.114178658**			
Ghi.7102.1.S1_s_at	Gorai.005G198500.1	VOZ	-0.749282047			
Ghi.4612.1.A1_s_at	Gorai.004G266000.1	Whirly				0.280008261

GhiAffx.39894.1.A1_at	Gorai.003G002200.1	WOX		0.736653634		0.765770961
Ghi.3743.1.A1_at	Gorai.009G124000.1	WRKY	**4.743463219**	**3.759452541**	**5.071424857**	**4.116592943**
Ghi.9953.2.S1_at	Gorai.008G253300.1	WRKY	**4.622164039**		**4.164907172**	**4.103697316**
GhiAffx.7683.2.A1_at	Gorai.001G002200.1	WRKY	**2.368606532**	**2.021631414**	**2.259313085**	**3.321742408**
GhiAffx.41278.1.S1_at	Gorai.011G041400.1	WRKY	**3.005722923**	**2.791226807**	**3.646218335**	**3.318202387**
Ghi.9192.1.S1_s_at	Gorai.009G062300.1	WRKY				**3.294052463**
Ghi.1132.1.A1_s_at	Gorai.004G134600.1	WRKY	**2.435636743**		**3.303935622**	**3.216490298**
Ghi.3273.1.A1_at	Gorai.010G182500.1	WRKY	**3.710427536**	**4.231223245**	**2.609297276**	**3.201633861**
GraAffx.28971.2.A1_at	Gorai.009G062300.1	WRKY			**2.748015588**	**3.184437818**
Ghi.9240.3.A1_s_at	Gorai.012G119600.1	WRKY	**2.571357577**		**3.692984508**	**3.075207486**
Ghi.1132.3.S1_s_at	Gorai.004G134600.1	WRKY	**2.598849534**		**2.591031166**	**2.899748526**
Ghi.10332.1.A1_x_at	Gorai.004G160100.1	WRKY	**3.10614363**	1.815512615	1.896465224	**2.798005597**
Ghi.3743.1.S1_at	Gorai.009G124000.1	WRKY	**4.11907692**	**3.245260035**	**3.179444685**	**2.795985341**
GraAffx.25344.1.S1_a_at	Gorai.010G118300.1	WRKY	**2.087369379**		**2.453905376**	**2.70523687**
GhiAffx.60347.1.S1_s_at	Gorai.004G160100.1	WRKY	**3.102504294**	1.973898554	1.836434409	**2.689519167**
Ghi.9568.1.S1_at	Gorai.010G222400.1	WRKY	**2.816118142**		**3.122474669**	**2.558542063**
Ghi.9240.1.S1_s_at	Gorai.012G119600.1	WRKY	**2.31267952**		**3.098580505**	**2.442532375**
Ghi.10061.1.S1_s_at	Gorai.010G118300.1	WRKY			**2.476653223**	**2.417167271**
Ghi.9466.3.A1_s_at	Gorai.003G150700.1	WRKY	**2.35453501**			**2.347427018**
Ghi.9182.3.S1_at	Gorai.011G012700.1	WRKY	1.990529403	1.562079231	**2.258057394**	**2.274264711**
GarAffx.5818.1.S1_s_at	Gorai.003G150700.1	WRKY	1.663307022		**2.458314684**	**2.268020028**
Ghi.2995.2.A1_at	Gorai.011G201800.1	WRKY	**2.127843313**	1.019305393	**2.081437427**	**2.18127869**
Ghi.1132.2.A1_a_at	Gorai.004G134600.1	WRKY	1.97896533			**2.130589353**
GhiAffx.30199.1.S1_at	Gorai.007G121200.1	WRKY		**3.772291453**		**2.096150677**
GhiAffx.5407.1.S1_at	Gorai.011G012700.1	WRKY		1.653963287	**2.000378835**	1.989694931
Ghi.10018.1.S1_s_at	Gorai.001G002200.1	WRKY	**2.055225853**		1.563332641	1.903821397
Ghi.9182.4.S1_at	Gorai.011G012700.1	WRKY	1.760962436	1.149362725	1.95089438	1.81094694
Ghi.9182.1.A1_s_at	Gorai.011G012700.1	WRKY			1.777761922	1.760900936
GhiAffx.8448.1.A1_at	Gorai.009G066900.1	WRKY	**2.704353109**		**2.064397104**	1.717511199

GhiAffx.34717.1.S1_at	Gorai.001G214800.1	WRKY	**2.746246968**	**3.308034779**	**2.770545316**	1.686852685
Ghi.3273.2.S1_at	Gorai.010G182500.1	WRKY	**3.026320252**	**2.412650631**	**2.372890498**	1.667327656
Ghi.9201.1.A1_at	Gorai.007G107300.1	WRKY			1.914280779	1.501613879
GraAffx.20526.1.A1_s_at	Gorai.012G119600.1	WRKY	**2.469236532**		1.336459954	1.372799043
GraAffx.30430.1.S1_s_at	Gorai.010G121800.1	WRKY			1.448545311	1.192473695
Ghi.2995.1.S1_at	Gorai.011G201800.1	WRKY				1.145974887
Ghi.3815.1.A1_at	Gorai.003G026600.1	WRKY	0.98392729		1.122343599	1.057772286
GhiAffx.1859.1.S1_at	Gorai.002G181600.1	WRKY				1.056703011
Ghi.8955.2.S1_s_at	Gorai.010G022600.1	WRKY			0.642954143	1.056649951
GhiAffx.6177.1.S1_at	Gorai.011G086300.1	WRKY	**2.442183898**		**2.625521006**	0.792323454
GhiAffx.25448.1.S1_at	Gorai.012G186000.1	WRKY				0.642750647
Ghi.3815.1.S1_at	Gorai.003G026600.1	WRKY		-0.570679736		0.486666044
Ghi.6768.1.A1_s_at	Gorai.013G008300.1	WRKY		**-2.147223575**		-1.672701535
Ghi.132.1.S1_s_at	Gorai.001G021500.1	WRKY		**-2.347965846**		**-2.156943835**
GhiAffx.5398.1.S1_s_at	Gorai.007G122300.1	WRKY		1.662549084		
Ghi.6771.1.A1_s_at	Gorai.007G122300.1	WRKY		1.354732822		
Ghi.3273.3.S1_at	Gorai.010G182500.1	WRKY		1.321205872		
Ghi.9953.2.A1_at	Gorai.008G253300.1	WRKY	**2.981297224**	1.198779864		
Ghi.6771.1.S1_at	Gorai.007G122300.1	WRKY		1.094279667		
Ghi.132.1.A1_s_at	Gorai.001G021500.1	WRKY		**-2.114065518**		
Ghi.3139.1.S1_at	Gorai.003G047800.1	WRKY		**-2.761080308**		
Ghi.9193.2.S1_s_at	Gorai.010G219200.1	WRKY		**-3.022255015**		
Ghi.3139.1.A1_at	Gorai.003G047800.1	WRKY		**-3.069900426**		
Ghi.9193.2.A1_at	Gorai.010G219200.1	WRKY		**-3.236145115**		
GhiAffx.2014.1.S1_s_at	Gorai.012G013900.1	WRKY		**-3.458293632**		
Ghi.9212.1.A1_at	Gorai.007G014600.1	WRKY	**3.874009118**			
Ghi.10018.2.A1_s_at	Gorai.001G002200.1	WRKY	1.584880308			
GhiAffx.25167.1.A1_at	Gorai.007G089700.1	YABBY			**-3.68010183**	**-2.39181119**
Gra.2731.1.S1_s_at	Gorai.009G201300.1	ZF-HD			-1.892347715	

Supplementary Table S7. *Helicoverpa armigera*-responsive stress-related genes. Ratio conidered significant ($q \leq 0.05$, $\log_2|\text{Ratio}| \geq 2$) are indicated in red (induced) or green (repressed).

Probe Set ID	Maping to *Gossypium raimondii* gene	Fold Change 6h (treatment/control)	Fold Change 12h (treatment/control)	Fold Change 24h (treatment/control)	Fold Change 48h (treatment/control)
GbaAffx.211.1.S1_s_at	Gorai.009G447600.1			**6.3799334**	**7.0775095**
GbaAffx.212.1.S1_at	Gorai.009G446800.1		**2.5982114**	**4.0949165**	**6.2118683**
GhiAffx.8053.1.A1_at	Gorai.003G083500.1	**5.3628602**	**5.1644777**	**6.0848568**	**5.7042395**
Ghi.102.1.S1_x_at	Gorai.009G428400.1		**4.9252662**	**6.6534949**	**4.8693098**
Ghi.8390.2.S1_x_at	Gorai.011G197300.1	**3.7047482**		**4.6592144**	**3.813089**
Ghi.1011.1.A1_s_at	Gorai.006G150200.1	**2.4831171**			**3.3478744**
GhiAffx.19307.1.S1_s_at	Gorai.009G085300.1	**3.6133117**	**5.0689983**	**2.0399044**	**3.2526012**
Ghi.10425.3.A1_s_at	Gorai.002G235900.1	**2.2579826**			**3.2397365**
Ghi.9232.1.S1_at	Gorai.003G140400.1				**3.1947836**
GbaAffx.201.1.S1_at	Gorai.012G129000.1	1.8085475		**2.8871177**	**3.1531148**
Ghi.10425.3.S1_s_at	Gorai.002G235900.1	**2.4615211**			**3.1278077**
Ghi.10228.1.S1_at	Gorai.007G132800.1		**2.2856217**	**3.0380148**	**3.0702667**
Ghi.6523.1.S1_s_at	Gorai.012G129000.1			**2.2593934**	**2.9128127**
Ghi.1011.2.A1_at	Gorai.006G150200.1	**2.8016941**		**2.0790396**	**2.8493738**
GbaAffx.201.1.S1_s_at	Gorai.012G129000.1			**2.133166**	**2.8211945**
GraAffx.31063.1.A1_at	Gorai.003G140400.1				**2.6875772**
GhiAffx.21120.1.A1_at	Gorai.006G184100.1	**3.7596627**	**2.4774074**	**2.7303637**	**2.602723**
GhiAffx.24798.1.S1_at	Gorai.006G185500.1		1.9753686	**2.1962371**	**2.5578829**
Ghi.8524.1.S1_at	Gorai.006G259500.1		**4.1206515**		**2.5173774**
Gra.1544.1.A1_s_at	Gorai.008G007200.1		1.5935449	**2.6142024**	**2.4271406**
GhiAffx.43038.1.S1_at	Gorai.008G065900.1	**3.353807**	**4.2442809**	**2.3661186**	**2.3843032**
GhiAffx.24905.1.S1_s_at	Gorai.007G304400.1		1.7310169		**2.309798**

Ghi.6876.1.A1_s_at	Gorai.013G202800.1	**3.492867**	**3.2921246**		**2.3032463**
GarAffx.6150.1.S1_at	Gorai.013G164900.1	**4.5448276**		**4.1907294**	**2.2366518**
GraAffx.25124.1.A1_at	Gorai.012G129000.1			1.5622623	**2.1707845**
GhiAffx.21657.1.S1_at	Gorai.005G062700.1			0.9507196	**2.1027137**
Ghi.7707.2.A1_s_at	Gorai.007G304400.1		1.4639545	1.0796011	**2.016857**
GhiAffx.40179.1.A1_at	Gorai.001G195900.1	1.6139638	**3.1616631**	**2.2296467**	1.9743326
GhiAffx.25235.1.A1_at	Gorai.003G163400.1	**2.0245454**		1.850743	1.7567001
Ghi.10826.1.S1_at	Gorai.006G079400.1			**4.2417893**	1.6330018
Gra.2150.1.S1_s_at	Gorai.010G215600.1	**2.2367032**		**2.2021389**	1.4606262
Ghi.5843.1.A1_s_at	Gorai.006G259500.1		**2.4323734**		1.4261801
GhiAffx.49963.1.S1_s_at	Gorai.013G164800.1	**2.9996629**		**2.8510347**	
GraAffx.27014.1.S1_at	Gorai.008G282100.1			**2.3764879**	
GraAffx.11740.1.A1_s_at	Gorai.008G065900.1	**2.1416476**	**4.5622752**		
Ghi.3337.1.A1_at	Gorai.011G019700.1	**3.9360221**	**4.0139537**		
Ghi.359.1.S1_at	Gorai.006G009100.1		**2.584007**		
GhiAffx.10611.1.S1_at	Gorai.009G045500.1		**2.4804059**		
Ghi.6462.1.S1_x_at	Gorai.011G197500.1	**2.8807848**		**3.6709743**	
Ghi.8360.1.A1_at	Gorai.007G348500.1			**3.2765708**	
Ghi.8071.1.S1_at	Gorai.011G197200.1	**3.061**	**2.0155042**	**3.2300895**	
Ghi.7942.2.A1_at	Gorai.008G029500.1	**2.1013728**	1.8752363	1.7656398	
Ghi.246.1.S1_s_at	Gorai.002G194500.1	**2.2439965**	**2.4353**		
Ghi.789.1.S1_at	Gorai.009G035500.1			**-7.1608715**	**-6.5419004**
Ghi.4513.1.A1_at	Gorai.002G161000.1			**-6.0094604**	**-6.3155442**
Gra.3119.1.A1_at	Gorai.009G035500.1			**-5.1840762**	
Gra.674.1.A1_at	Gorai.002G161000.1			**-4.934219**	**-5.3168685**
GhiAffx.21879.1.S1_at	Gorai.004G101200.1			**-4.1484605**	**-3.168335**
Ghi.4241.1.S1_at	Gorai.002G219200.1			**-3.411454**	-1.9638225
GhiAffx.60328.1.S1_at	Gorai.004G101300.1			**-3.2549733**	**-3.7156531**

Ghi.335.1.S1_at	Gorai.003G015700.1	**-2.3753668**	-1.439715	**-3.0680194**	**-2.8274075**
Gra.2405.1.A1_at	Gorai.N010400.1			**-2.889173**	
Ghi.7551.1.S1_at	Gorai.003G101000.1			**-2.7358767**	**-2.3076934**
Ghi.459.1.S1_at	Gorai.005G184600.1			**-2.7126433**	**-2.7403635**
Ghi.8835.1.A1_x_at	Gorai.010G049900.1			**-2.709225**	
Ghi.6410.1.S1_at	Gorai.N010400.1			**-2.697907**	
Ghi.6309.2.A1_at	Gorai.003G140500.1	-1.0447101		**-2.2088823**	-1.6137192
GhiAffx.24344.1.S1_at	Gorai.011G263500.1	**-2.7988831**	**-2.6137937**	-1.8017485	
GraAffx.11047.3.A1_s_at	Gorai.009G200600.1				**-2.8681062**
GhiAffx.50774.1.S1_s_at	Gorai.004G105500.1				**-2.6368044**
Ghi.9795.1.S1_s_at	Gorai.009G200600.1				**-2.5878676**
GraAffx.16056.1.S1_s_at	Gorai.001G082300.1				**-2.1243455**
GhiAffx.49772.1.S1_at	Gorai.010G228600.1		**-2.2807059**		
Ghi.6899.1.A1_at	Gorai.008G274600.1		**-2.5269164**		
GhiAffx.3815.1.A1_s_at	Gorai.008G274600.1		**-2.5512309**		
Ghi.3844.1.S1_at	Gorai.009G143900.1		**-2.7007622**		
Gra.1079.1.A1_s_at	Gorai.011G116900.1				1.6660769
Gra.2907.1.A1_at	Gorai.008G260000.1		1.7589027		1.6266788
Gra.3118.1.A1_s_at	Gorai.011G064400.1				1.2989463
GhiAffx.22348.1.A1_s_at	Gorai.007G045800.1				1.2107752
Ghi.6433.1.S1_at	Gorai.008G044000.1				1.159175
Gra.2602.1.A1_s_at	Gorai.011G013000.1	1.9846626		1.0868249	0.9907981
GhiAffx.43317.1.A1_s_at	Gorai.004G244100.1		1.7856111		0.9717157
GraAffx.9152.1.S1_at	Gorai.003G163400.1				0.8907662
Ghi.7578.1.S1_x_at	Gorai.011G013000.1	1.5539972		1.0379799	0.8693292
Ghi.5742.1.A1_s_at	Gorai.008G177700.1				0.6283644
Ghi.10319.1.S1_s_at	Gorai.009G208400.1			0.8861907	0.5468326
GbaAffx.2.1.S1_at	Gorai.004G237800.1				0.4628114

Ghi.6382.1.S1_s_at	Gorai.013G206300.1	-0.8856472			0.3931017
GhiAffx.31795.1.A1_s_at	Gorai.001G083800.1	-0.6980718			-0.3264061
Ghi.5185.1.A1_s_at	Gorai.001G083800.1	-0.6201287		-0.6831622	-0.5000854
GraAffx.20558.1.A1_s_at	Gorai.006G088100.1		0.8572641		-0.5079753
Ghi.9656.2.S1_at	Gorai.009G044700.1				-0.5791399
GraAffx.32178.1.A1_a_at	Gorai.007G304100.1				-0.6812903
Gra.1023.1.A1_at	Gorai.003G040000.1	-0.6793644			-0.7148498
GhiAffx.7854.1.A1_at	Gorai.013G163600.1	-1.2903089		-0.8653993	-0.7251991
GhiAffx.24606.1.S1_a_at	Gorai.005G239100.1	-1.8068965		-1.0594284	-0.7438932
GhiAffx.20332.1.S1_at	Gorai.001G137800.1	-1.3997849		-0.869669	-0.7838949
GhiAffx.27140.1.S1_s_at	Gorai.003G055000.1	-1.0063716			-0.8551598
GhiAffx.43839.1.S1_at	Gorai.004G096000.1	-1.8025143		-1.2691069	-0.9575922
Ghi.5625.1.A1_s_at	Gorai.008G025200.1			-1.762089	-0.9646346
Gra.49.1.S1_at	Gorai.007G093200.1			-1.3543684	-1.0323157
Ghi.9656.2.A1_s_at	Gorai.009G044700.1			-1.4136659	-1.0881876
Ghi.9683.1.S1_s_at	Gorai.010G008900.1				-1.1181486
Gra.783.1.S1_s_at	Gorai.001G083800.1	-0.9269168	-1.0269834	-1.0195385	-1.2102488
GraAffx.27481.1.S1_at	Gorai.001G137800.1	-1.2319718	-0.8537474	-1.3685886	-1.2207882
GhiAffx.25836.1.S1_at	Gorai.005G112500.1	-1.5255249		-1.5160926	-1.2280423
Ghi.10345.1.S1_at	Gorai.005G239400.1		-1.3219264		-1.2843445
GraAffx.32888.2.S1_s_at	Gorai.010G008900.1				-1.523255
Ghi.6181.1.S1_s_at	Gorai.004G234000.1	-1.8645448		-1.5525965	-1.6204808
Gra.2949.1.S1_s_at	Gorai.004G234000.1	-1.7801433		-1.5557541	-1.6427047
GraAffx.32888.1.A1_at	Gorai.010G008900.1				-1.8533542
Gra.2085.2.S1_s_at	Gorai.009G124300.1	1.3460049		1.3177102	
Gra.2150.1.A1_s_at	Gorai.010G215600.1	1.2204381		0.902183	
Gra.2451.1.S1_x_at	Gorai.009G105500.1			0.7105808	
GbaAffx.2.1.S1_x_at	Gorai.004G237800.1	-0.8855349		-0.6509256	

Ghi.3991.2.A1_s_at	Gorai.006G259800.1	-0.8217319		-0.6859319	
GbaAffx.2.1.S1_s_at	Gorai.004G237800.1	-0.9333974		-0.7480712	
Ghi.1591.1.S1_s_at	Gorai.004G237800.1	-1.1620924		-0.8693429	
Gra.2694.1.S1_s_at	Gorai.011G179700.1	-1.6401451		-1.1379554	
GhiAffx.87.1.A1_at	Gorai.009G282000.1			-1.2915587	
Ghi.7518.3.S1_x_at	Gorai.013G093600.1			-1.7021767	
Ghi.7518.1.S1_s_at	Gorai.013G093600.1			-1.7913255	
Ghi.7533.2.S1_at	Gorai.005G236100.1			-1.9193454	
GhiAffx.10455.1.S1_s_at	Gorai.008G090400.1		1.8851854		
GraAffx.11047.1.A1_a_at	Gorai.009G200600.1	1.9316597			
Ghi.3357.2.A1_s_at	Gorai.012G028400.1	1.7953385			
Ghi.4935.1.S1_s_at	Gorai.001G078000.1	1.6755173			
GhiAffx.19330.2.S1_at	Gorai.006G035800.1	1.3153779			
Ghi.3357.1.S1_s_at	Gorai.012G028400.1	1.2990177			
Ghi.10319.2.S1_s_at	Gorai.009G208400.1	0.9496034			
GhiAffx.4850.1.S1_s_at	Gorai.009G291100.1	-0.731539			
GhiAffx.8135.1.S1_s_at	Gorai.013G135100.1	-0.8554548			
Ghi.5908.1.A1_s_at	Gorai.009G069500.1	-0.9941146			
Ghi.3442.3.S1_x_at	Gorai.007G171700.1	-1.0679084			
GhiAffx.26244.1.A1_s_at	Gorai.002G062100.1	-1.1422101			
GhiAffx.33426.1.S1_at	Gorai.004G097500.1	-1.1578819			
GraAffx.34176.1.A1_s_at	Gorai.013G206300.1	-1.1863733			
Ghi.3442.3.A1_at	Gorai.007G171700.1	-1.2724561			
Ghi.3442.2.A1_x_at	Gorai.007G171700.1	-1.3040755			
Ghi.7942.2.S1_a_at	Gorai.008G029500.1	1.7628224	1.4910103	1.8576758	1.6553864
Ghi.8364.1.A1_at	Gorai.004G090800.1	1.5511491			1.5182416
Ghi.9169.1.S1_s_at	Gorai.008G193500.1	1.989533			1.514631
Ghi.10639.1.S1_s_at	Gorai.009G189500.1				1.3860463

GraAffx.30479.1.A1_a_at	Gorai.009G189500.1				1.216386
Ghi.10735.1.S1_s_at	Gorai.004G089400.1		0.99401		1.0550019
GraAffx.14181.1.A1_s_at	Gorai.004G089400.1	0.823439	1.1503981	0.848076	0.9360922
Ghi.3390.4.S1_at	Gorai.007G321000.1	1.7796193			0.8443902
Ghi.925.1.S1_at	Gorai.007G318500.1				0.8369386
Ghi.1885.1.S1_s_at	Gorai.002G130700.1	1.5917376		1.0873993	0.7790236
Gra.724.1.A1_s_at	Gorai.009G430200.1				-0.4638838
GhiAffx.22009.1.S1_at	Gorai.011G220700.1	-1.5049183	-1.5037364		-0.6220511
GhiAffx.24809.1.S1_at	Gorai.007G290200.1				-0.6434384
GhiAffx.26707.1.A1_at	Gorai.013G108200.1				-0.7859249
GhiAffx.30928.1.S1_at	Gorai.007G344000.1				-0.8543348
Ghi.6443.2.A1_s_at	Gorai.013G200400.1			1.8922133	
GhiAffx.34653.1.S1_s_at	Gorai.013G165600.1	1.6181152		0.9145162	
Ghi.4946.1.S1_s_at	Gorai.010G035500.1		0.659467		
Gra.2927.1.S1_s_at	Gorai.012G099600.1		-1.0343533		
Ghi.6496.1.S1_a_at	Gorai.004G090700.1	1.847824			
Ghi.8619.1.A1_at	Gorai.007G319900.1	1.4791495			
Ghi.3390.1.S1_at	Gorai.007G321000.1	1.2972384			
Ghi.6496.3.A1_at	Gorai.004G090700.1	1.1602133			
GhiAffx.40129.1.A1_at	Gorai.007G185200.1	0.7556219			
Ghi.1106.1.S1_at	Gorai.011G263000.1	-0.9729426			
GhiAffx.20784.1.A1_at	Gorai.011G068500.1				-0.9869709
GhiAffx.24133.1.S1_at	Gorai.013G248100.1	1.0761625			0.7055121
GhiAffx.24500.1.S1_at	Gorai.008G251200.1	-1.6631177		-1.7062959	-1.2662837
GhiAffx.40178.1.S1_x_at	Gorai.009G447600.1			1.8426449	
Ghi.1407.1.A1_s_at	Gorai.001G100700.1	1.6826537			1.7313498
Ghi.3750.2.A1_at	Gorai.009G373300.1				0.5388292
Ghi.3391.2.S1_at	Gorai.007G319200.1		-0.9930294		

Gra.2013.4.S1_s_at	Gorai.009G199700.1				-0.6304416

Supplementary Table S8. Overall summary of *Helicoverpa armigera*-responsive genes involved in secondary metabolite pathways.

	Number of genes involved in phytohormone biosynthesis				
Category	$q \leq 0.05$	$q \leq 0.05$, $\log_2$|Ratio| ≥ 2	up	down	mix
Phenylpropanoid	48	28	16	12	0
Flavonoid	37	23	2	21	0
Terpenoid	61	22	16	6	0

Supplementary Table S9. Subset of *Helicoverpa armigera*-responsive genes involved in secondary metabolite pathways.

Secondary metabolism	Probe Set ID	Maping to *Gossypium raimondii* gene	Fold Change 6h (treatment/control)	Fold Change 12h (treatment/control)	Fold Change 24h (treatment/control)	Fold Change 48h (treatment/control)
Phenylpropanoid	Ghi.4039.1.A1_s_at	Gorai.002G248000.1				**-2.2438331**
	Gra.2239.1.S1_s_at	Gorai.002G248000.1				**-2.7037683**
	Gra.2682.2.S1_s_at	Gorai.002G248000.1				**-3.2065204**
	Ghi.3957.2.S1_at	Gorai.009G416300.1	**2.9105658**			
	Ghi.9347.1.S1_s_at	Gorai.011G207000.1				0.7024802
	GarAffx.30221.1.S1_s_at	Gorai.011G207000.1		0.7243949		-1.1097273
	Gra.2891.1.S1_s_at	Gorai.011G207000.1	**2.8444294**			-1.1271584
	Ghi.4426.1.S1_s_at	Gorai.013G271700.1				-1.1383155
	Gra.2823.1.S1_s_at	Gorai.013G271700.1	1.7426937			-1.2446286
	GarAffx.24670.1.S1_s_at	Gorai.013G271700.1				-1.8106341
	GarAffx.24670.1.S1_at	Gorai.013G271700.1	**2.4345656**			
	Gra.2967.2.S1_s_at	Gorai.013G271700.1	**3.17718**			
	Ghi.1797.1.S1_at	Gorai.011G053700.1		1.1757043	1.7532559	1.3511112
	Gra.787.2.A1_s_at	Gorai.003G052100.1			**2.1838396**	1.0433396
	Ghi.9697.1.A1_s_at	Gorai.003G052100.1				**-2.1363479**
	Ghi.9697.2.S1_s_at	Gorai.003G052100.1		-1.7499572	-1.3125971	**-2.352277**
	GhiAffx.6286.1.S1_at	Gorai.009G005900.1				**-2.3659901**
	GhiAffx.36401.1.S1_at	Gorai.009G148700.1				**-2.8979617**
	Ghi.8716.1.A1_at	Gorai.009G148700.1			**2.0659803**	
	Ghi.4973.1.A1_s_at	Gorai.009G122600.1	**2.2269293**		**2.4480471**	1.4239488
	GhiAffx.3584.1.S1_s_at	Gorai.004G264500.1	**-2.4904775**		-1.9226274	-1.0494909
	Ghi.9525.2.S1_s_at	Gorai.009G122600.1	**2.5378976**		-1.698525	-1.5800164
	Ghi.8197.1.S1_at	Gorai.011G130700.1			-1.9178507	-1.8481037
	GhiAffx.44780.1.S1_at	Gorai.011G130700.1	-1.2044231		**2.6822168**	

	GraAffx.15192.1.A1_s_at	Gorai.013G000900.1	**2.4008102**		**2.1247702**	1.1308033
	GhiAffx.16334.1.S1_s_at	Gorai.013G000900.1	**2.3423648**		1.8319279	0.9202353
	Ghi.4349.1.A1_at	Gorai.N022200.1	**2.1575033**	**3.3187674**	**3.7074213**	**3.32197**
	Gra.1442.1.S1_s_at	Gorai.008G024000.1	**2.7504863**	1.1061073	1.7362072	1.1466652
	Gra.1442.2.A1_s_at	Gorai.008G024000.1			0.8975983	
	Ghi.1309.1.S1_s_at	Gorai.008G024000.1	**2.0284313**			
	Ghi.3490.1.S1_a_at	Gorai.012G149800.1	**2.6834946**		**2.7509323**	1.645233
	GraAffx.15192.1.A1_s_at	Gorai.013G000900.1	**2.4008102**		**2.1247702**	1.1308033
	GhiAffx.16334.1.S1_s_at	Gorai.013G000900.1	**2.3423648**		1.8319279	0.9202353
	GraAffx.16056.1.S1_s_at	Gorai.001G082300.1				**-2.1243455**
	GhiAffx.20332.1.S1_at	Gorai.001G137800.1	-1.3997849		-0.869669	-0.7838949
	GraAffx.27481.1.S1_at	Gorai.001G137800.1	-1.2319718	-0.8537474	-1.3685886	-1.2207882
	GhiAffx.27140.1.S1_s_at	Gorai.003G055000.1	-1.0063716			-0.8551598
	GhiAffx.50774.1.S1_s_at	Gorai.004G105500.1				**-2.6368044**
	Ghi.6181.1.S1_s_at	Gorai.004G234000.1	-1.8645448		-1.5525965	-1.6204808
	Gra.2949.1.S1_s_at	Gorai.004G234000.1	-1.7801433		-1.5557541	-1.6427047
	Gra.2085.2.S1_s_at	Gorai.009G124300.1	1.3460049		1.3177102	
	GhiAffx.4850.1.S1_s_at	Gorai.009G291100.1	-0.731539			
	GhiAffx.20332.1.S1_at	Gorai.001G137800.1	-1.3997849		-0.869669	-0.7838949
	Ghi.1871.1.S1_at	Gorai.007G231800.1	1.3443482		0.9517879	
	Ghi.1607.1.S1_s_at	Gorai.008G022800.1			0.7536121	0.5922738
	Ghi.7475.1.S1_s_at	Gorai.008G262100.1		-0.6872649		
	Gra.798.1.S1_s_at	Gorai.009G192400.1	0.7850365			**-2.0627712**
	Gra.2877.1.A1_at	Gorai.009G192400.1				**-2.121138**
Terpenoid	GhiAffx.58844.1.S1_at	Gorai.011G184200.1	-1.7465257		-1.5868971	-0.71619
	GraAffx.24921.1.A1_s_at	Gorai.011G184200.1			-0.8243968	
	Ghi.2066.1.A1_s_at	Gorai.004G111100.1	-1.0634509			
	GraAffx.27434.1.S1_s_at	Gorai.006G189200.1		1.0074544		-0.5183396

	Ghi.7742.1.S1_s_at	Gorai.006G189200.1		1.1222758		
	GraAffx.27887.1.S1_s_at	Gorai.010G134700.1				-0.6447989
	GhiAffx.24261.1.S1_s_at	Gorai.010G134700.1				-0.6662388
	Ghi.204.1.S1_s_at	Gorai.008G243100.1	1.4373867		**3.3925222**	**2.8012229**
	Ghi.306.4.S1_s_at	Gorai.005G215500.1	**2.0703858**	**2.0371405**	**2.3117842**	1.8011008
	GraAffx.17247.3.A1_s_at	Gorai.005G215800.1			**-2.971463**	**-2.107727**
	Ghi.306.1.S1_s_at	Gorai.005G215500.1	0.8386151	**2.0856181**	1.2517312	
	Ghi.306.2.S1_at	Gorai.005G215800.1		1.8473614	0.9432924	
	GraAffx.17247.2.S1_s_at	Gorai.005G215800.1		1.7545547		
	Gra.1436.4.A1_s_at	Gorai.005G215800.1		1.7395707		
	GhiAffx.61130.1.S1_at	Gorai.013G065200.1		-0.6872012		
	Ghi.9008.2.S1_at	Gorai.007G342800.1			1.3368917	1.2636103
	Ghi.9008.1.A1_s_at	Gorai.007G342800.1			1.2881508	0.7987058
	Ghi.3344.2.A1_x_at	Gorai.008G223300.1	0.9044795		1.3894006	1.0516027
	Ghi.3344.1.S1_s_at	Gorai.008G223300.1			1.3015535	0.8985383
	Ghi.6469.1.S1_s_at	Gorai.013G012400.1	1.2730784		1.5124836	1.248351
	Ghi.6497.1.S1_at	Gorai.013G012400.1	1.4571384			0.7357546
	Ghi.8010.1.S1_s_at	Gorai.004G262800.1	0.9918467	1.024844		0.9237855
	Ghi.8010.3.A1_at	Gorai.004G262800.1	0.9750133			0.7235433
	Ghi.8010.2.S1_s_at	Gorai.004G262800.1				0.6938014
	GhiAffx.19330.2.S1_at	Gorai.006G035800.1	1.3153779			
	GraAffx.9456.1.S1_s_at	Gorai.011G047900.1	**-2.4422618**	-1.9935578	**-2.3304906**	-1.9566151
	Ghi.3310.1.A1_s_at	Gorai.011G047900.1	-1.276653			
	Ghi.3337.1.A1_at	Gorai.011G019700.1	**3.9360221**	**4.0139537**		
	Ghi.359.1.S1_at	Gorai.006G009100.1		**2.584007**		
	GhiAffx.19330.2.S1_at	Gorai.006G035800.1	1.3153779			
	Ghi.3549.2.S1_s_at	Gorai.009G184900.1	**2.0683928**			
	GraAffx.1932.1.S1_at	Gorai.009G184900.1	1.9992871			

	Gra.1135.2.A1_a_at	Gorai.009G184900.1	1.5513999			
	GhiAffx.22628.1.A1_s_at	Gorai.009G184900.1	1.4142556			
	Ghi.101.1.S1_s_at	Gorai.008G094500.1				-0.3072293
	Ghi.4897.1.A1_at	Gorai.013G052900.1		0.9575984	0.856481	0.6059668
	GhiAffx.21567.1.S1_at	Gorai.005G228100.1	-0.8152308			0.3824628
	Ghi.102.1.S1_x_at	Gorai.009G428400.1		**4.9252662**	**6.6534949**	**4.8693098**
	GarAffx.37202.1.S1_x_at	Gorai.009G428500.1			**3.5407909**	**2.2595394**
	GarAffx.6150.1.S1_at	Gorai.013G164900.1	**4.5448276**		**4.1907294**	**2.2366518**
	GhiAffx.49963.1.S1_s_at	Gorai.013G164800.1	**2.9996629**		**2.8510347**	
	Ghi.6637.2.S1_at	Gorai.011G102700.1		**3.0951666**		0.6293606
	Ghi.8644.1.S1_at	Gorai.007G051300.1	-1.1090274	-1.1124154	-0.6694657	-0.7344949
	Ghi.6888.1.S1_at	Gorai.003G097600.1	**-2.3236799**	**-2.0435143**	**-2.1695607**	-1.2577783
	Gra.2631.1.A1_s_at	Gorai.003G097600.1		-1.3182184	**-2.708894**	-1.9185348
	Ghi.4.2.S1_at	Gorai.002G237300.1			**4.2408796**	**3.0983291**
	Ghi.4.1.A1_at	Gorai.002G237200.1				**2.6590376**
	GraAffx.27948.1.S1_s_at	Gorai.006G151900.1	**-2.5284659**		-0.8674729	
	GhiAffx.13394.2.S1_x_at	Gorai.004G187100.1		-1.394983		
	GhiAffx.12577.1.S1_at	Gorai.001G047500.1				1.4117924
	GraAffx.15730.1.S1_at	Gorai.006G005500.1	**-3.3160196**		-1.626301	-0.6493604
	Ghi.9280.3.A1_at	Gorai.013G028600.1		**2.2074117**		
	Ghi.8028.1.S1_a_at	Gorai.001G173000.1	-1.6491532			
	GhiAffx.4691.1.A1_at	Gorai.002G038900.1	**4.3621167**			
	Ghi.1172.3.A1_at	Gorai.001G212500.1	1.3557291	**3.6258788**	**2.3219674**	**2.8745244**
	Ghi.8983.2.S1_at	Gorai.001G170500.1	-1.5564502			
	Ghi.8983.1.S1_at	Gorai.001G170500.1	-1.8043258			
	GraAffx.31069.1.A1_s_at	Gorai.001G170600.1				-1.0967881
	Ghi.8452.1.S1_s_at	Gorai.007G097200.1				-1.2205284
	GhiAffx.42319.1.A1_at	Gorai.011G134500.1				-1.3355786

	GhiAffx.25014.1.S1_at	Gorai.011G229300.1	-1.9040732	-0.5594301	-0.8810036	-0.6218277
Flavonoid	Gra.2508.1.A1_x_at	Gorai.011G161300.1				**-2.1085901**
	Gra.2508.2.S1_x_at	Gorai.011G161200.1				**-2.1133961**
	Ghi.1443.1.S1_x_at	Gorai.011G161300.1				**-2.5306507**
	GraAffx.25565.2.S1_s_at	Gorai.011G161200.1				**-2.8494854**
	Ghi.10134.3.S1_s_at	Gorai.005G035100.1				**-2.856162**
	Gra.207.1.A1_s_at	Gorai.005G035100.1				**-3.9211315**
	Ghi.10760.1.S1_s_at	Gorai.013G023400.1				-1.8274856
	Gra.48.1.A1_a_at	Gorai.013G023400.1				**-2.2071397**
	GraAffx.33125.1.A1_at	Gorai.012G014600.1				**-2.9588453**
	GarAffx.21877.1.S1_s_at	Gorai.012G014600.1				**-3.3972535**
	Gra.2794.2.S1_s_at	Gorai.007G194700.1				**-2.9425959**
	Gra.2007.1.S1_x_at	Gorai.008G062900.1				**-3.3967302**
	Ghi.2175.1.S1_s_at	Gorai.001G134900.1				**-2.4605086**
	Gra.605.2.A1_x_at	Gorai.001G134900.1				**-2.733115**
	Gra.2986.2.S1_at	Gorai.008G239200.1	-0.979027			-0.6736186
	Ghi.4416.1.A1_x_at	Gorai.008G198200.1				-1.4295998
	Gra.968.1.S1_at	Gorai.008G198200.1	0.7942168			-1.7849009
	Gra.297.1.A1_at	Gorai.008G284200.1				**-2.3704344**
	Ghi.2175.1.S1_s_at	Gorai.001G134900.1				**-2.4605086**
	Ghi.6547.1.S1_s_at	Gorai.003G165800.1		1.5866036	**4.252102**	
	Ghi.318.1.S1_at	Gorai.007G151200.1		-1.8610472		
	GraAffx.33404.1.A1_at	Gorai.009G121600.1			-1.1782218	-0.7088383
	Ghi.9683.1.S1_s_at	Gorai.010G008900.1				-1.1181486
	GraAffx.11047.1.A2_at	Gorai.009G200600.1	**-2.4548835**	-0.8827628	-1.569613	-1.2400758
	GraAffx.32888.2.S1_s_at	Gorai.010G008900.1				-1.523255
	GraAffx.32888.1.A1_at	Gorai.010G008900.1				-1.8533542
	Ghi.9795.1.S1_s_at	Gorai.009G200600.1				**-2.5878676**

	GraAffx.11047.3.A1_s_at	Gorai.009G200600.1				**-2.8681062**
	GhiAffx.10611.1.S1_at	Gorai.009G045500.1		**2.4804059**		
	GraAffx.11047.1.A1_a_at	Gorai.009G200600.1	1.9316597			
	Ghi.1234.1.S1_s_at	Gorai.004G205900.1				**-3.0941811**
	GhiAffx.4183.1.S1_s_at	Gorai.009G175500.1				**-3.3896122**
	GraAffx.21935.1.S1_s_at	Gorai.009G175500.1				**-4.1585799**
	GraAffx.28822.1.S1_at	Gorai.002G250500.1	-1.1019455			-0.4352485
	GhiAffx.24031.1.S1_at	Gorai.002G250500.1	-1.7841454	-1.4592238		
	Ghi.6535.1.A1_at	Gorai.012G071800.1	-0.9830021			
	Gra.2610.1.S1_s_at	Gorai.012G071800.1	-1.0328634			

Supplementary Table S10. Primers used for real time qPCR.

**Target gene**	**Forward (5’→3’)**	**Reverse (5’→3’)**
Ghi.3957.2.S1_at	AGCGGCTAAGAAGTTGCATG	TTCGATCTGTGGACCAAGCC
GhiAffx.8460.1.S1_at	TACTCGGTCGCTAACTTGGC	CGGCAAGCTCAAGAAAACGT
Gra.2995.1.S1_s_at	TGTTGTCCCTGGAAGCTGTC	TGGTCGAGCAACAAATCCGA
GhiAffx.43038.1.S1_at	AATACACACCATCGCCAGCA	GCACTTGGCGACGTTGTAC
Gra.2794.2.S1_s_at	TGCCCACAACCTGATCTCAC	TTGTCCCTGGTGGCTTGAAG
Ghi.1234.1.S1_s_at	CACCATCGAGATCCTCAGCA	AGGAGGGAACAGTGGAGGTT
Ghi.204.1.S1_s_at	TTGCCCCTGAGAAGTTCGTC	ACCAGTACAGTTCCCGTCCT
Ghi.306.4.S1_s_at	AGGTTGGTACTGTTGGTGGTG	CGTTTGCTCCTGGTGTCTCT
GraAffx.27887.1.S1_s_at	TGGGAAATGCAGACTCCACA	ACAGGATGCTTAAGGTGCTCC
Ghi.6469.1.S1_s_at	TCTCTGAGCTGCAAAAGTGTCA	AGGATTCAAACCTCAATTGGACA
GhiAffx.58844.1.S1_at	TGGAGGCCATTGGTTCTTCC	AGCACATTGAACACCGTTGC
Ghi.3549.2.S1_s_at	GGTTGAACGGATGCTGGACT	ACCGAGCGCACTTGTAAGAA
GhiAffx.45408.1.S1_s_at	CGTGACCCTGCAATTTTCCG	CGCGCCTCATTTTCACAACA
Ghi.102.1.S1_x_at	AGCATGTTGAGAGTGCTTGGA	GATTCCACCCTTAGCCGCTT
Ghi.359.1.S1_at	GGCCGCTAAGTCTCTTTCGT	ACGCTCTCTCCTTAGGCTCA
GhACT4	TCGCTCGTTCCTTGTATAATTCATC	TGGATTCCAGCAGCTTCCA
GhPP2A1	CACCGTCTGCGGCGATA	CCACCGATGCGAAACAGTT
